# NGS Evaluation of Colorectal Cancer Reveals Interferon Gamma Dependent Expression of Immune Checkpoint Genes and Identification of Novel IFNγ Induced Genes

**DOI:** 10.3389/fimmu.2020.00224

**Published:** 2020-03-19

**Authors:** Lai Xu, Lorraine Pelosof, Rong Wang, Hugh I. McFarland, Wells W. Wu, Je-Nie Phue, Chun-Ting Lee, Rong-Fong Shen, Hartmut Juhl, Lei-Hong Wu, Wei-Lun Alterovitz, Emanuel Petricon, Amy S. Rosenberg

**Affiliations:** ^1^Office of Oncologic Diseases, Center for Drug Evaluation and Research (CDER), FDA, Silver Spring, MD, United States; ^2^Office of Biotechnology Products, Division of Biotechnology Review and Research III (DBRRIII), Office of Pharmaceutical Quality (OPQ), Center for Drug Evaluation and Research (CDER), FDA, Silver Spring, MD, United States; ^3^Facility for Biotechnology Resources, Center for Biologics Evaluation and Research (CBER), FDA, Silver Spring, MD, United States; ^4^Indivumed GmbH, Hamburg, Germany; ^5^Division of Bioinformatics and Biostatistics (DBB), National Center for Toxicological Research (NCTR), FDA, Jefferson, AR, United States; ^6^HIVE, Center for Biologics Evaluation and Research (CBER), FDA, Silver Spring, MD, United States; ^7^Center for Applied Proteomics and Molecular Medicine (CAPMM), George Mason University, Fairfax, VA, United States

**Keywords:** colorectal cancer, IFNγ gradient, immune checkpoint genes, co-expression network, novel immune checkpoint related genes

## Abstract

To evaluate the expression of immune checkpoint genes, their concordance with expression of IFNγ, and to identify potential novel ICP related genes (ICPRG) in colorectal cancer (CRC), the biological connectivity of six well documented (“classical”) ICPs (CTLA4, PD1, PDL1, Tim3, IDO1, and LAG3) with IFNγ and its co-expressed genes was examined by NGS in 79 CRC/healthy colon tissue pairs. Identification of novel IFNγ- induced molecules with potential ICP activity was also sought. In our study, the six classical ICPs were statistically upregulated and correlated with IFNγ, CD8A, CD8B, CD4, and 180 additional immunologically related genes in IFNγ positive (FPKM > 1) tumors. By ICP co-expression analysis, we also identified three IFNγ-induced genes [(IFNγ-inducible lysosomal thiol reductase (IFI30), guanylate binding protein1 (GBP1), and guanylate binding protein 4 (GBP4)] as potential novel ICPRGs. These three genes were upregulated in tumor compared to normal tissues in IFNγ positive tumors, co-expressed with CD8A and had relatively high abundance (average FPKM = 362, 51, and 25, respectively), compared to the abundance of the 5 well-defined ICPs (Tim3, LAG3, PDL1, CTLA4, PD1; average FPKM = 10, 9, 6, 6, and 2, respectively), although IDO1 is expressed at comparably high levels (FPKM = 39). We extended our evaluation by querying the TCGA database which revealed the commonality of IFNγ dependent expression of the three potential ICPRGs in 638 CRCs, 103 skin cutaneous melanomas (SKCM), 1105 breast cancers (BC), 184 esophageal cancers (ESC), 416 stomach cancers (STC), and 501 lung squamous carcinomas (LUSC). In terms of prognosis, based on Pathology Atlas data, correlation of GBP1 and GBP4, but not IFI30, with 5-year survival rate was favorable in CRC, BC, SKCM, and STC. Thus, further studies defining the role of IFI30, GBP1, and GBP4 in CRC are warranted.

## Introduction

CRC is the second leading cause of cancer-related mortality in the United States^1^ (1) and, disturbingly, an increased incidence of CRC in patients <40 years of age has been reported (2). In recent years, immunotherapeutic approaches have opened important treatment options in a small subset of CRC patients with microsatellite instability high (MSI-H) tumors (3). Most CRC, however, are microsatellite stable (MSS) (4). In MSI-H CRC patients, the high response rate to the ICP blockade appears due to a higher tumor mutational burden, the presence of neoantigens and consequent infiltration by CD8^+^ (T_C_, cytotoxic T lymphocyte) CTL and higher expression levels of ICPs (5). In this regard, IFNγ has been identified as the lynchpin factor in the induction and sustained expression of ICPs on tumor and infiltrating T cells in several tumor types and thus, qPCR detection of IFNγ has been considered a potential marker of response to ICP blockade in several cancer studies including in non-small cell lung cancer (NSCLC) and cutaneous melanoma (SKCM) (6–8). However, the role of IFNγ in establishing the immunological profile of CRC has not been thoroughly investigated. This prompted us to use NGS to evaluate expression of IFN-γ in CRC tumors, its link to known IFNγ-dependent ICPs, and to identify novel ICPRGs. In this study, we evaluated expression levels of six well-known immune checkpoint genes [six ICPs (CTLA4, PD1, PDL1, Tim3, IDO1, and LAG3)] as well as potential immune checkpoint related genes (ICPRGs) also induced by IFNγ by next generation sequencing (NGS) in 79 stringently collected and preserved primary human CRCs and their patient matched normal colonic tissues. Expression levels of six ICPs were evaluated as were their relationships to expression levels of IFNγ and other immunologically pertinent genes. Based on the ICP co-expression network, we searched for potential ICP related genes (ICPRGs) in IFNγ positive tumors that may function as novel ICPs and consequently identified IFI30, GBP1 and GBP4. Based on the identified literature (9–22), IFI30, GBP1, and GBP4 suppress mouse primary T cell activation *in vitro* and mouse innate immune response *in vivo* while IFI30 and GBP1 appear to increase cell proliferation in a glioma cell line and two breast cancer cell lines but diminish cell proliferation in a colon cancer cell line. Intriguingly, however, IFI30 RNA expression is associated with better patient survival in breast cancer (12) and diffuse large B cell lymphomas (DLBCL) (14) while GPB1 RNA is associated with better patient survival in melanoma (20) but poorer prognosis in human glioblastoma (21).

## Materials and Methods

### Cohort

Seventy-nine paired-tissues (79 tumor and 79 normal controls, Table S1) of pretreatment CRCs were collected from 38 male and 41 female patients by Indivumed GmbH (Germany) for mRNA sequencing. The purchase of these de-intified samples was exempted by FDA IRB/RIHSC. To evaluate tumor content, hematoxylin and eosin stained microscopic slices were examined by pathologists to determine the tumor cell and normal cell areas, respectively. Histologically, tumor samples had 50–70% content of cancer cells while normal samples had 0% content of cancer cells. Normal tissues were collected from a site at a minimum of 5 cm from the tumor margin. Ischemia time was 6–11 min. This short cold ischemia reduces post-surgical tissue processing artifacts (23). According to the medical pathology reports, tumors were classified as well, moderately, and poorly differentiated tumors following international guideline UICC TNM-classification (24). For the convenience of analysis, 26 stage I and II tumors were considered as low stage tumors (LSTs), while 53 stage III and IV tumors were considered as HSTs (25). In this study, a normal control adjacent to a low stage tumor is referred to as LSN. The ratio of high stage tumors vs. low stage tumors is 2–1. Among 26 low stage tumors, there were two either lymph node (LN) or lymphatic vessel (LV) positive tumors while among 53 high stage tumors, there were 28 either LN/LV positive tumors. For tumor grades, there were 17 well (low grade) differentiated, 36 moderately (medium grade) differentiated, and 26 poorly (high grade) differentiated tumors. Clinical and histopathological characteristics of the patients as well as tumor location are summarized in Table S1. Among these 80 tumor pairs, 79 pairs were sequenced (all except the T7/N7 pair). The information for the cohort of 50 CRC tumor pairs, 588 CRCs, 103 SKCMs, 1105 BCs, 184 ESCs, 416 STCs, and 501 LUSC for validation of six ICPs and three ICPRGs was extracted from TCGA_B38 through OncoLand (Tables S2–S4). As for tumor stage information of validating cohort, there were 57 LST and 82 HSTs (Tables S1, S2).

For protein and survival data, The Clinical Proteomic Tumor Analysis Consortium (CPTAC) (https://cptac-data-portal.georgetown.edu/) which contains Mass spectroscopy (MS) analyses of 95 CRCs (Table S5) and The Pathology Atlas (https://www.proteinatlas.org/humanproteome/pathology) were used.

### mRNA Sequencing

RNA quality was assessed using the Agilent 2100 Bioanalyzer, with cellular RNA analyzed using the RNA 6000 Nano Kit (Agilent). Samples with an RNA Integrity Number (RIN) of 7 or higher were processed to generate libraries for mRNA sequencing following the Illumina® TruSeq Stranded mRNA Sample Preparation Guide. In this method, poly-A mRNAs were purified from 0.5 μg total RNA, fragmented and reverse-transcribed into cDNAs. Double strand cDNAs were adenylated at the 3′ ends and ligated to indexed sequencing adaptors, followed with amplification for 15 cycles. One femtomole of the sequencing libraries (median size ~260 nt) were denatured and loaded onto a flow cell for cluster generation using the Illumina cBot. Every six samples were loaded onto each lane of a rapid run flow cell. Paired-end sequencing was carried out on a HiSeq 2500 sequencer (Illumina, San Diego, CA, USA) for 100 × 2 cycles (26). For each sample, we obtained ~50 million 100-bp reads that passed preset filtering parameters (27).

### Sequencing Data Analysis

For mRNA sequencing, Tophat V.2.0.11 was used to align reads in fastq files to the UCSC human hg19 reference genome. Cufflinks V.2.2.1 was used to assemble the transcriptome based on the hg19 reference annotation, and Cuffquan/Cuffnorm (part of Cufflinks) were used in calculating relative abundance of each transcript reported as FPKM. Gene co-expression analyses were carried by Partek NGS & microarray data analysis software (25, 28, 29). Integrated Discovery (DAVID) v6.7 (https://david.ncifcrf.gov/) was used for biological pathway determination and Cytoscape (2.8.2) was used for gene co-expression networks construction.

### Initial Expression Landscape of CRC

A total of 25,761 genes were detected. Because genes with higher FPKM values may have greater biological impact, we focused on genes with FPKM > 1 (25, 28). Ten thousand two hundred fifty-five genes (40% of total genes) had an average FPKM > 1 and differential expression between tumors and normal controls (False Discovery Rate (FDR) < 0.05 in ANOVA). A total of 3,893 genes (15% of total genes) with average FPKM > 1 showed no differential expression between tumor and normal controls with FDR (ANOVA) > 0.05 (25, 29).

### NGS Evaluation of Immune Gene Expression

NGS is a technology that accurately quantifies gene expression and does not necessarily require further validation, as supported by the literature (25). To more fully establish NGS as a “stand alone” technology for gene quantification, we reasoned that NGS quantification of a critical hub gene should be reflected in the consequent up/downregulation of highly interconnected genes and thus examined the co-expression of IFNγ genes with T and NK cell specific genes based on the fact that IFNγ and granzymes are produced by T cells and NK cells (30). IFNγ was highly correlated (cc > 0.80) with 9 classical T and NK cell gene markers and two granzymes in IFNγ positive tumors, as assessed by NGS: CD8A [0.97], CD69 [0.93], CD52 [0.86], CD160 [0.85], CD3E [0.84], CD96 [0.83], CD8B [0.82], CD2 [0.82], CD7 [0.80], GZMA [0.80], and GZMM [0.80]) (Table S6). These data indicate that NGS expression profiles of immune related genes do not necessarily require validation by other gene quantification technologies, especially for hypothesis-generating studies.

## Results

### Upregulation of Six Established ICPs Associated With Higher Expression of IFNγ in CRC

**Figure 1:**
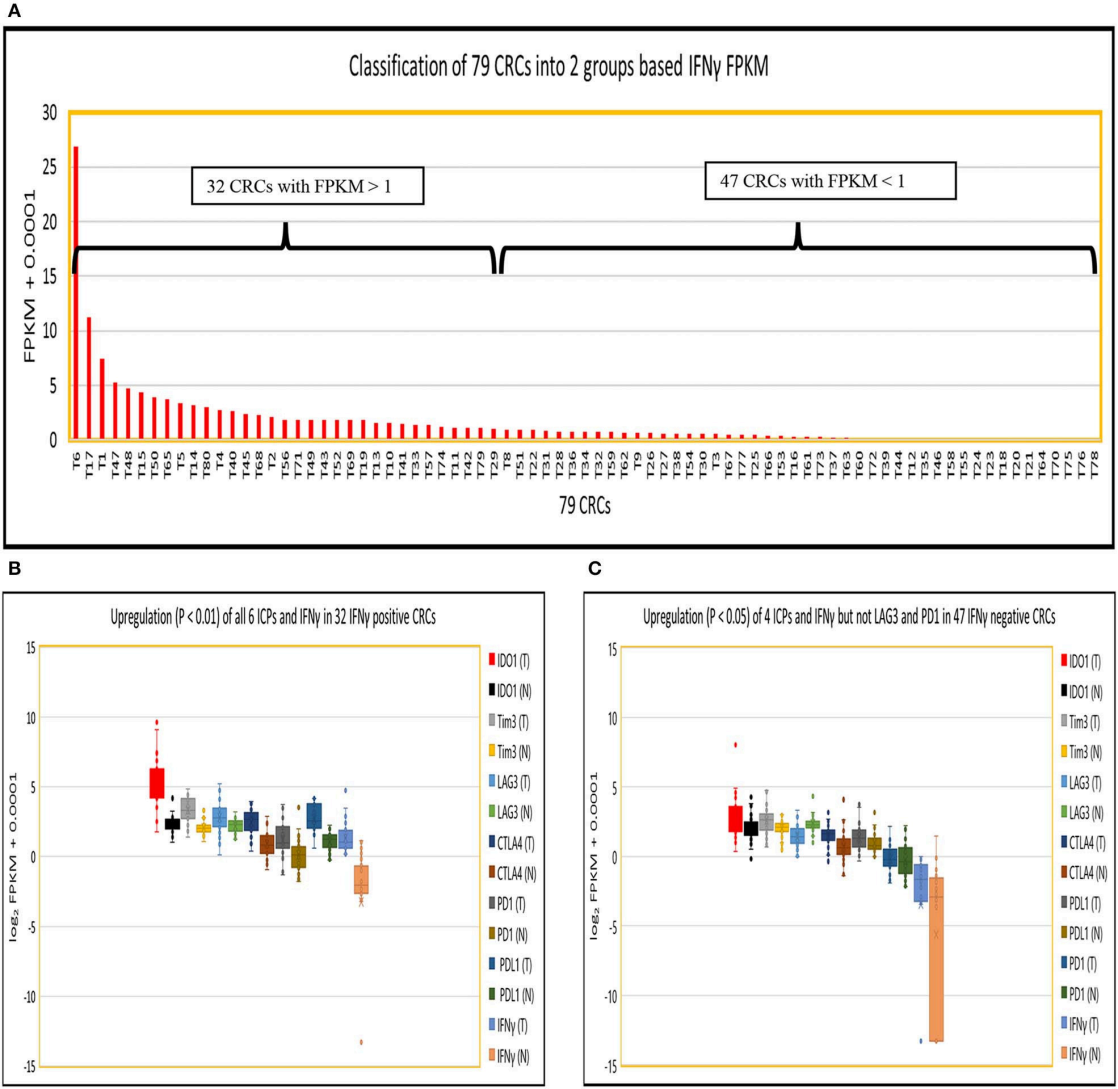
Expression of IFNγ in 79 CRC pairs. **(A)** Subtyping of 79 CRCs into CRC with high IFNγ (FPKM > 1) and CRC with low IFNγ (FPKM < 1). **(B)** Box and Whisker plot of six ICPs in INFγ positive CRC. More upregulation of all six ICPs and IFNγ (IDO1, 7.8-fold; Tim3, 2.3-fold, LAG3, 1.6-fold; CTLA4, 2.9-fold; PDL1, 3.0-fold; PD1, 2.1-fold; and IFNγ, 24.1-fold) in tumor vs. normal (*P* < 0.01). **(C)** Box and Whisker plot of six ICPs in INFγ negative CRC. Less upregulation (*P* < 0.05) of four ICPs and IFNγ (IDO1, 1.4-fold; Tim3, 1.4-fold; CTLA4, 1.7-fold; PDL1, 1.3-fold; and IFNγ, 4.4-fold), downregulation of LAG3 (0.54-fold) (*P* = 1.7E-05), and no dysregulation of PD1 (1.0-fold) (*P* = 0.57) in tumor vs. normal.

Because IFNγ has been strongly implicated in the induction of PDL1 expression in tumors, and PD1 expression in tumor infiltrating T cells (6–8), we divided 79 CRCs into those with potentially significant IFNγ expression (abundance level of FPKM > 1; 32 CRCs), designated IFNγ^+^ (positive), and those expressing lower levels of IFNγ expression (FPKM < 1; 47 CRCs), designated IFNγ^−^ (negative) (Figure 1A). The log_2_ FPKM plot of tumor and normal showed that IFNγ and all six well-documented ICPs were significantly upregulated (*p* < 0.01) in IFNγ positive CRCs compared to their patient matched normal tissue controls [Figure 1B: IFNγ (24.1-fold), IDO1 (7.8-fold), CTLA4 (2.9-fold), Tim3 (2.3-fold), PDL1 (3.0-fold), PD1 (2.1-fold) and LAG3 (1.6-fold)] while only 4 ICPs were significantly upregulated (P < 0.05) in IFNγ negative CRCs compared to their matched controls (Figure 1C): IFNγ (4.4-fold), IDO1 (1.4-fold), CTLA4 (1.7-fold), Tim3 (1.4-fold), PDL1 (1.3-fold), and PD1 (1.0-fold). Intriguingly, LAG3 (0.54-fold) was significantly downregulated (*p* = 1.7E-0.5) in IFNγ negative CRCs (Figure 1C). These data suggest that differential expression of ICPs, especially LAG3, may pertain to levels of IFNγ expression in CRCs. Regarding the quantitative relationship between IFNγ and the six ICPs, the expression levels of these six were 1.9 to 6.4 -fold higher in IFNγ positive vs. IFNγ negative tumors (Table S7), though even in the IFNγ positive tumors, these ICPs were expressed at relatively low abundance (average FPKM = 3–12) compared to oncogenes such as MYC, CDK4, and CCND1 (average FPKM = 105) (25), with the exception of IDO1 which is robustly upregulated (average FPKM = 89). These data suggest a positive effect of IFNγ with respect to consequent upregulation of ICPs but potentially at levels still insufficient to promote significant expression of ICP proteins on tumor and infiltrating T cells in CRC, supporting the lack of response of most of these tumors to ICP inhibitor therapeutics.

### ICP Co-expression Profile in IFNγ Positive and Negative Tumors

**Figure 2:**
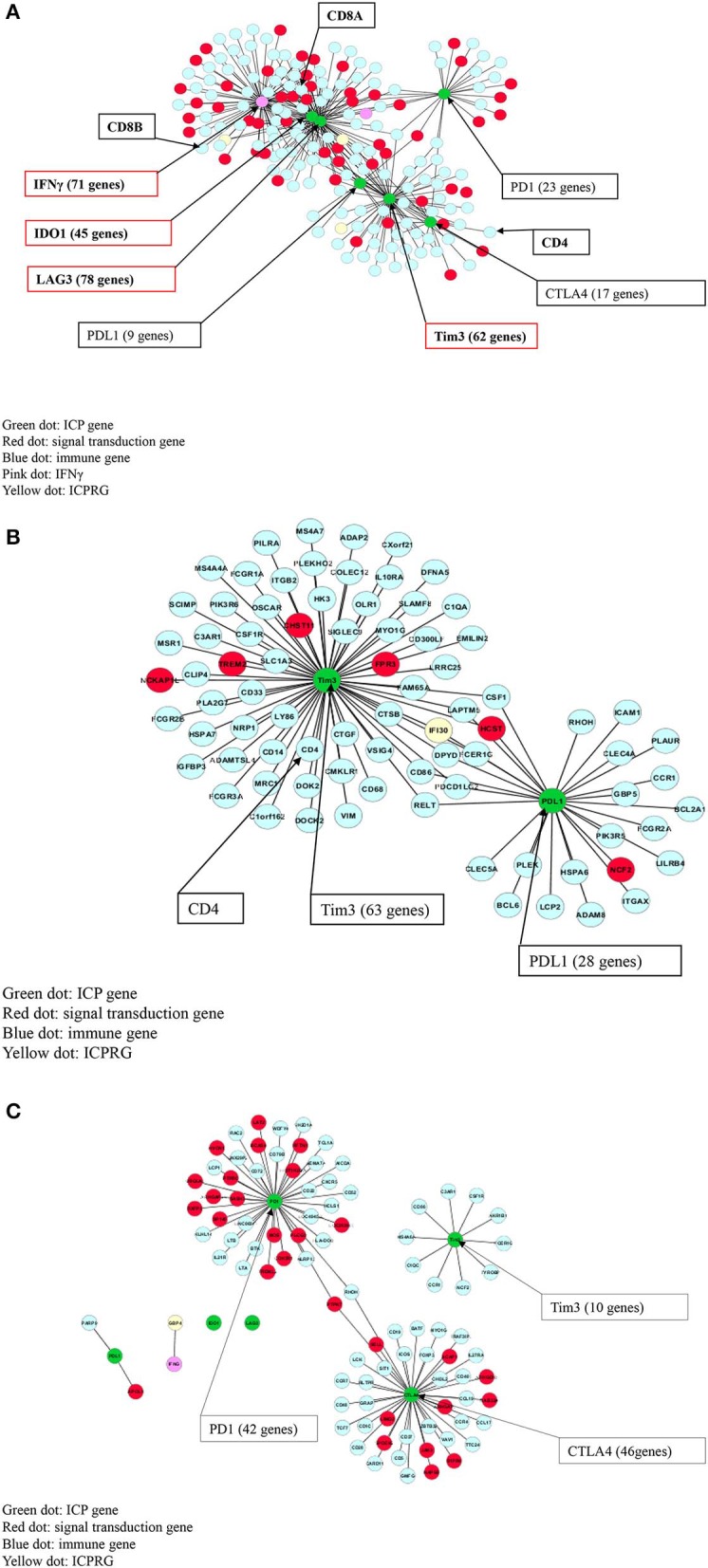
Co-expression (cc > 0.8) of IFNγ with six ICPs in CRC. **(A)** IFNγ and all six ICPs co-expressed with CD8A/CD8B/CD4 within one 190 gene network in 32 IFNγ positive CRCs. **(B)** Tim3 and PDL1 co-expressed with CD4 within one 83 gene network in 47 IFNγ negative CRCs. **(C)** CTLA4 co-expressed with PD1 within one 88 gene expression network, but not Tim3, in normal colon without CD8/CD4.

We then performed a Pearson correlation analysis to evaluate the co-expression profile of IFNγ and the six ICPs in IFNγ positive and negative CRC, as well as in normal controls using a stringent correlation coefficient (cc) > 0.8. In IFNγ positive CRC, the following was observed: (i) IFNγ, CD8A, CD8B, and CD4 co-expression with all six ICPs genes within one network (190 genes); (ii) IFNγ and three ICPs (LAG3, Tim3, and IDO1) were defined as potential hub genes due to their substantial number of co-expressed genes (*n* > 45); and (iii) co-expression of IFNγ and six ICPs with 129 immune cell related genes (pale blue dots in Figure 2A) and 54 signaling genes (red dots in Figure 2A; Table S8).

In contrast, in IFNγ negative CRC, co-expression of Tim3, PDL1, and CD4 was found within one network comprised of 83 genes, but without linkage to IFNγ or other immune checkpoint genes including PD1, CTLA4, IDO1 and LAG3. Genes co-expressed with Tim3 and PDL1 consisted of 77 immune related genes (pale blue dots in Figure 2B) as well as six signaling genes (red dots in Figure 2B; Table S8).

In control normal colonic tissues, co-expression of two ICPs (CTLA4 and PD1) was observed but also was not linked to IFNγ and CD4, CD8A, and CD8B, or co-expression with PDL1, Tim3, LAG3, and IDO1. However, expression of CTLA4 and PD1 was noted within an 88-gene network including 63 immune related genes (pale blue dots in Figure 2C) as well as 25 signaling genes (red dots in Figure 2C; Table S8).

### Identification of Three Novel ICPRG Genes in CRC

**Figure 3:**
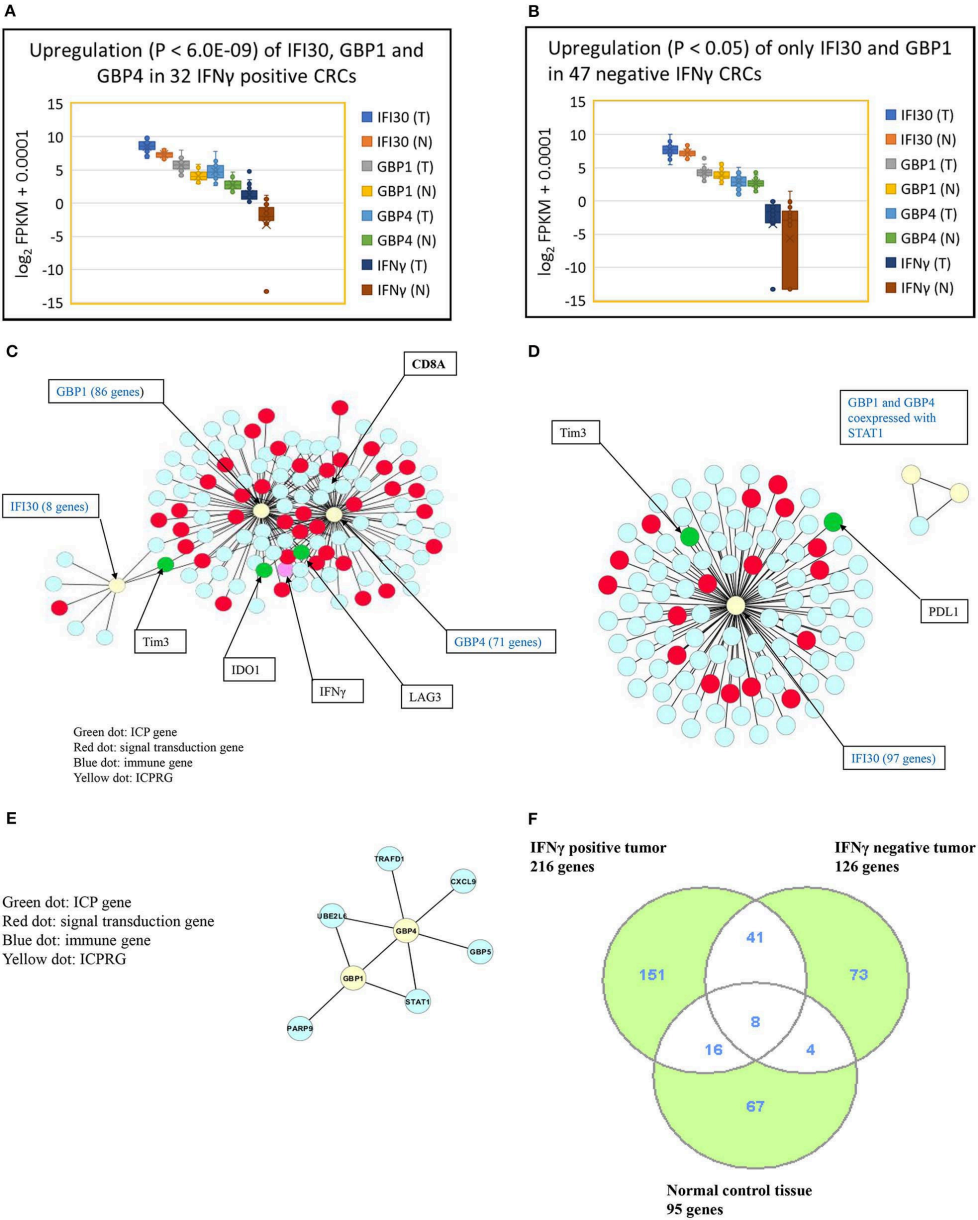
Characterization of three ICPRGs in CRC. **(A)** Box and Whisker plot of three ICPRGs in IFNγ positive CRC. More upregulation (*P* < 6.0E-09) of all three ICPRGs (IFI30: 2.3-fold; GBP1: 3.1-fold, GBP4; 12.9-fold in tumor vs. normal). **(B)** Box and Whisker plot of three ICPRGs in IFNγ negative CRC. Less upregulation (*P* < 0.05) of two ICPRGs (IFI30: 1.4-fold and GBP1: 1.2-fold) and GBP4 (1.1-fold) (*P* = 0.23) in tumor vs. normal. **(C)** Co-expression of IFI30, GBP1, and GBP4 with IFNγ, IDO1, Tim3, LAG3 and CD8A within a 119 gene network in IFNγ positive CRCs. **(D)** Co-expression of IFI30 with PDL1 and Tim3 within a 101 network without CD8/CD4 in IFNγ negative CRCs. **(E)** GBP1 co-expressed with GBP4 within an eight-gene network in normal colon. **(F)** Identification of unique genes co-expressed with 10 genes (INFγ, six ICPs, and three ICPRGs) between INFγ positive and negative tumors. There are 151 unique genes in IFNγ positive tumors, 73 unique genes in IFNγ negative tumors, and 67 unique genes in normal tissue.

We next explored whether there were potential novel ICPRGs from the IFNγ co-expression network that potentially factored into the refractoriness of CRC to immunotherapy. Three genes, IFI30, GBP1, and GBP4, were identified by two criteria: co-expressed (cc > 0.8) with known ICPs and upregulated by a minimum 2-fold average over normal (T/N). In IFNγ positive tumors, IFI30, GBP1, and GBP4 were significantly upregulated (*p* < 0.0001) at 2.7-, 4.2-, and 6.2-fold, respectively (Figure 3A) while only IFI30 and GBP1 were upregulated (p < 0.05) at 1.4- and 1.2-fold, respectively in IFNγ negative tumors compared to their matched normal controls (Figure 3B). Notably, the abundance of IFI30, GBP1 and GBP4 was substantially higher in IFNγ positive tumors (362, 51, 25 FPKM, respectively) than in IFNγ negative tumors (207, 18, 7 FPKM, respectively) (Table S9). These three genes have documented immune suppressive function and pro or anti-tumor roles in different types of cancers (9–23).

We then employed a Pearson correlation analysis to study the co-expression of IFNγ with the three newly identified potential ICPRGs in IFNγ positive and negative tumors as well as in normal controls. In IFNγ positive tumors, all three novel ICPRGs were co-expressed with IFNγ/CD8A and three known ICPs (IDO1, Tim3, and LAG3) within one network (total 119 genes: GBP1 and GBP4 correlated with IDO1, LAG3, and CD8A; IFI30 correlated Tim3) (Figure 3C). Moreover, GBP1 and GBP4 were identified as potential hub genes due to their substantial number of co-expressed genes (*n* > 70). In IFNγ negative tumors, IFI30 was co-expressed with 97 genes including Tim3 and PDL1 (but not with CD8/CD4) while GBP1 and GBP4 were only co-expressed with STAT1 (signal transducer and activator of transcription 1) but not with IFI30 or with IFNγ (Figure 3D). In normal colonic tissue, GBP1 and GBP4, were co-expressed within an 8 gene network, including STAT1 and GBP5 but not in association with IFNγ or IFI30 (Figure 3E), though IFI30 is strongly expressed in normal tissue (Table S9).

To address whether protein expression correlates with quantification of identified genes, we took advantage of two critical databases: Clinical Proteomic Tumor Analysis Consortium and The Pathology Atlas. The Clinical Proteomic Tumor Analysis Consortium CPTAC) (https://cptac-data-portal.georgetown.edu/) contains mass spectroscopy (MS) analyses of a cohort of 95 CRCs. By analysis of these data, we found that IFI30, GBP1, and GBP4 proteins are more abundantly expressed than are IDO1 and PD1 in this 95 CRC cohort (Figure S1A) (data pertaining to PDL1, Tim3, LAG3, CTLA4, and IFNγ are not available from this database) which is consistent with our findings that IFI30, GBP1, and GBP4 mRNAs (Table S9) were more abundant than six classical ICPs in our Indivumed and TCGA cohorts (Table S7).

Regarding the Pathology Atlas (https://www.proteinatlas.org/humanproteome/pathology), comprised of 5 million immunohistochemistry (IHC) images of different types of cancer, we compared the IHC data of the three novel ICP related genes and 5 classical checkpoint genes (ICPs) (CTLA4 is not available in this database) in CRC, breast cancer (BC), stomach cancer (STC) and skin cutaneous melanoma (SKCM). While 5 classical ICP proteins had low percentage staining (5–11%), the three ICP related proteins had high percentage staining (60–78%) in CRC and three other types of cancer (Figure S1B). This conclusion supports our findings that the proteins pertaining to the novel ICP related genes (Table S9), are not only markedly upregulated but are much more abundantly expressed than are the classical ICPs (Table S7) within tumor tissue, thus strongly supporting our gene expression data.

To gain insight into potential function of such genes in CRC, we sequenced six colon cancer cell lines (HCT15, SW480, SW620, SW116, HT29, HCT116, Colo205) and two normal colon cell lines (CCD841, HCoEpiC) and found that all 8 cell lines (both tumor and normal) had low expression of these three ICP related genes, IFNγ and six classical ICPs compared to primary tumors and normal tissues in HCA and PCA analyses (Figures S2A,B). The data again emphasize key differences between cell lines and primary tumors and that future functional studies, such as RNA silencing or over-expression of IFI30, GPB1, and GBP4 should be performed in primary tumors to evaluate the function of such factors in the tumor microenvironment context.

### Identification of Uniquely Co-expressed Genes in IFNγ Positive vs Negative Tumors as Well as Normal Control Tissues

Because IFNγ, the six ICPs and the three ICPRGs were co-expressed with different numbers of genes in IFNγ positive and negative tumors, as well as in normal controls, we identified non-overlapping as well as overlapping genes among these groups (Figures 2A–C, 3C–F) and consequently determined their related pathways with David Bioinformatics (25, 29). The results demonstrate that (i) in IFNγ positive tumors, 10 ICP and ICPRG genes (IFNγ, six ICPs and three ICPRGs) were uniquely co-expressed with 151 genes mainly related to CD8 T cell activation, inactivation, cytotoxicity, co-stimulation, response to vitamin A (T_H_ differentiation) and Wnt/β-catenin signaling (T cell development); (ii) in IFNγ negative tumors, three ICP and ICPRG genes (PDL1, Tim3, and IFI30) were uniquely co-expressed with 73 genes mainly related to B cell and macrophage activation, B cell antigen presentation, B cell response to lipopolysaccharide, wound healing/B cell maturation and EGFR signaling/B cell differentiation; (iii) in normal controls, 5 ICP and ICPRG genes (PD1, CTLA4, Tim3, GBP1, and GBP4) were uniquely co-expressed with 67 genes mainly related to T cell inhibition/MDSC2, lymph node development, induction of apoptosis, B cell proliferation and endoplasmic reticulum signaling/cell cycle (Tables 1A–C, Table S8). These data suggest the possible presence of distinct ICP/ICPRG involved in pathological and physiological pathways among IFNγ positive and negative tumors (two CRC subtypes) as well as in normal colonic tissues.

Table 1IFNγ dosage dependent immune checkpoint gene related pathways.

**Table:** 

**3 different groups**	**Total genes**	**Immune genes**	**Signaling genes**
**(A) NUMBER OF UNIQUELY CO-EXPRESSED GENES WITH SIX ICPs AND THREE ICPRGs IN IFNγ** **POSITIVE TUMOR, NEGATIVE TUMOR, AND NORMAL CONTROL**			
32 IFNγ positive CRCs	151	87	64
47 IFNγ negative CRCs	73	62	11
79 normal controls	67	52	15

**Table:** 

**4 main pathways identified from 87 unique immune genes co-expressed with** six **ICPs and three ICPRGs in IFNγ** **positive CRC**	**Pathway related genes**
**(B) IMMUNE PATHWAYS AMONG IFNγ** **POSITIVE TUMOR, NEGATIVE TUMOR, AND NORMAL CONTROL**	
CD8 T cell activation and inactivation (17 genes)	IFNγ, IDO1, LAG3, ITGAL, MICB, CD3G, CD3D, CD8A, CD8B, CD3E, SLA2, IL15, ADA, NLRC3, CD2, SPN, CD7
Cytolysis (7 genes)/T cell	DNASE2, GZMM, IL2RA, GZMA, GPR65, BIRC3, SRGN
T cell co-stimulation (2 genes)	TNFSF13B, SPN
Response to vitamin A (3 genes)/TH differentiation	CD38, MICB, MAP1B
**4 main pathways identified from 62 unique immune genes co-expressed with six ICPs and** three **ICPRGs in IFNγ** **negative CRC**	**Pathway related genes**
B cell and macrophage activation (7 genes)	ICAM1, PLEK, OLR1, CTGF, ITGA5, CD209, ADAM8
B cell antigen presentation (5 genes)	HCK, FCGR1A, FCER1G, COLEC12, CD14
B cell response to lipopolysaccharide (2 genes)	SLC11A1, PTAFR
Wound healing (6 genes)/B cell maturation	SLC11A1, PLEK, ITGA5, ANXA5, PLAU, PLAUR
**4 main pathways identified from 52 unique immune genes co-expressed with six ICPs and** three **ICPRGs in normal control**	**Pathway related genes**
T cell inhibition/MDSC2 (8 genes)	CD48, ZBTB32, CARD11, LCK, FOXP3, VAV1, LCP1, CD28, CCR7
Lymph node development (3 genes)/B cell	CXCR5, LTB, LTA
Induction of apoptosis (2 genes)/B cell	VAV1, CD5
B cell proliferation (2 genes)	CARD11, CD40, CD19, CD79B
**4 main pathways identified from 64 unique signaling genes co-expressed with six ICPs and three ICPRGs in IFNγ** **positive CRC**	**Pathway related genes**
**(C) SIGNALING TRANSDUCTION PATHWAYS AMONG IFNγ** **POSITIVE TUMOR, NEGATIVE TUMOR, AND NORMAL CONTROL**	
Wnt/β^−^catenin signaling (3 genes)/T cell development	NMI, RNF213, RNF31
GTPase signaling (6 genes)/T cell activation	GNGT2, GPR171, GPR174, GPR18, NCF1, SMAP2
Nuclear receptor signaling (13 genes)/T cell response	ATXN7, BTN3A2, CEP170, CSTF2, CTRL, FAM78A, GTF2H4, NPL, RFX5, SFMBT2, SMCHD1, SNTB2, SNX20
Phosphorylation (8 genes)/T cell activation	EVL, GSG2, HSPA1A, PPP1R16B, PTPN22, TBC1D10C, USF1, ZAP70
**4 main pathways identified from 11 unique signaling genes co-expressed with six ICPs and three ICPRGs in IFNγ** **negative CRC**	**Pathway related genes**
EGFR signaling (1 genes)/B cell differentiation	EMP3
Cell-cell recognition (1 genes)/B cell receptor	ST3GAL6
Phosphorylation (3 genes)/B cell receptor	ETV5, FGR, KIFC3
Ca2+ signaling (1 genes)	ITPRIP
**4 main pathways identified from 15 unique signaling genes co-expressed with six ICPs and three ICPRGs in normal control**	**Pathway related genes**
ER signaling (1 genes)/cell cycle	UBQLN3
Phospholipase (1 genes)/B cell receptor	PLCG2
Relaxin-3/RXFP3 signaling (1 genes)	RXFP3
TREM2/DAP12 signaling (1 genes) myeloid cell	TREM2

### Close Association of IFNγ With T Cell Gene Expression in IFNγ Positive CRC

**Figure 4:**
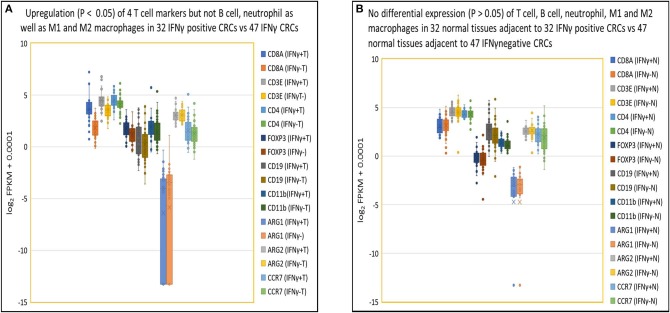
Close association of IFNγ with T cells in tumor but not in normal tissues. **(A)** Box and Whisker plot of immune cell genes in IFNγ positive CRCs. Upregulation (*P* < 0.05) of T cells (CD8A, 3.5-fold; CD3E, 2.0-fold; CD4, 1.3-fold; FOXP3, 1.5-fold) but not B cell (CD19) (1.2-fold, *P* = 0.38), neutrophil (CD11b) (1.4-fold, *P* = 0.079), M1 (ARG1) (0.68-fold, *P* = 0.62), (ARG2) (1.0-fold, *P* = 0.87), or M2 (ARG2, CCR7) (1.2-fold, *P* = 0.30) leukocyte-related genes in IFNγ positive CRC vs. negative CRC. **(B)** Box and Whisker plot of immune cell genes in normal colon tissue. No upregulation (*P* > 0.05) of T cells (CD8A, 0.93-fold; CD3E, 1.0-fold; CD4, 1.0-fold; FOXP3, 1.1-fold), B cell (CD19) (1.4-fold), neutrophil (CD11b) (1.1-fold), MDSC1 (ARG1) (1.0-fold), (ARG2) (0.97-fold), or MDSC2 (ARG2, CCR7) (1.2-fold) leukocytes related genes in tissues adjacent to IFNγ positive and negative CRCs.

As indicated in our evaluation of NGS for gene quantification (see Methods above), in IFNγ positive tumors, higher expression levels of IFNγ correlated specifically with higher expression levels of T cell related genes including CD8α (3.5-fold), CD3ε (2.0-fold), CD4 (1.3-fold), and FOXP3 (1.5-fold) compared to IFNγ negative tumors (*p* < 0.05) but not with expression levels of genes pertaining to other immune cells (*p* > 0.05), including B cells (CD19), neutrophils (CD11b), M1 macrophages (ARG1 and ARG2), and M2 macrophages (ARG2 and CCR7) (Figure 4A). In contrast, there were no differences (*p* > 0.05) in expression of the above 9 immune cell specific genes in normal tissues from patients with IFNγ positive vs. negative CRC (Figure 4B). As for T cell related cytotoxins, essential in tumor killing, stacked FPKM of 8 genes (PRF1, GZMM, GZMK, GZMH, GZMB, GZMA, FASLG, and FAS) demonstrated that these genes were more highly expressed 1.7-fold (*P* = 0.037) in IFNγ positive tumors compared to IFNγ negative tumors (Figure S3A). Eleven co-stimulatory genes (C10orf54 [B7-H5], BTLA4, CD86, CD80, ICOS, CD28, CD27, CD40, TNFRSF9 [4-1BB], TNFRSF18 [GITR], TNFRSF4 [OX40]) (30, 31) were expressed at higher (1.5-fold) levels in IFNγ positive vs. IFNγ negative CRC, but this did not reach statistical significance (*p* = 0.30) (Figure S3B). Moreover, stacked FPKM of 9 caspases (CASP2 to CASP10) (Figure S3C) and 9 cell cycle related genes (PRKDC, H2AFX, FANCD2, BRCA2, BRCA1, CHEK1, ATR, ATM) (Figure S3D) demonstrated that these genes were not significantly upregulated in IFNγ positive vs. negative tumors. These findings suggest that although IFNγ may upregulate CTL-associated proteins, it does not directly affect expression of co-stimulatory molecules (required for full T cell activation), caspases and cell cycle related genes, critical factors contributing to T cell activation and function.

In exploring further correlates of immune activation in tumors, we examined the relationship between IFNγ expression and thirteen DNA mismatch repair enzymes (MMR) (PMS2P5, PMS2P4, PMS2P3. PMS2P1, PMS2CL, PMS2, PMS1, MSH6, MSH4, MSH3, MSH2, MLH3, and MLH1), because the loss of DNA MMR function is associated with increased expression of neoantigens, immune cell recruitment and induction of ICPs (32). We did not find a difference between IFNγ expression and expression of 13 DNA MMRs (1.0-fold) (*p* = 0.86) in IFNγ positive vs. negative tumors (Figure S3E). Although the MSS/MSI status was available for only 7 CRCs in our cohort, 5 MSS CRCs, and two MSI CRC (Table S1), we found that all 10 genes (IFNγ, six ICPs, and three ICPRGs) were upregulated in MSI CRC compared to MSS CRC but this did not reach statistical significance (*P* = 0.31) in this small sample size (Figure S3F). Thus, the relationship between IFNγ upregulation and the presence or loss of the MMRs and MSI/MSS status needs to be studied further.

### Validation of IFNγ Dependent Expression of Six Classical ICPs and Three ICPRGs in a Larger CRC Cohort

**Figure 5:**
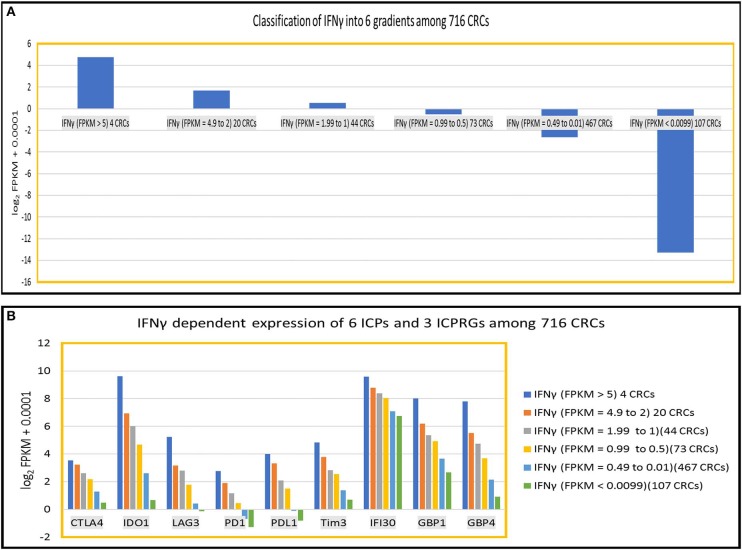
IFNy dependent expression of six ICPs (PDl, PDLl, CTLA4, IDOl, LAG3, Tim3) and three ICPRGs (IFI30, GBPl, GBP4) in 716 CRCs. **(A)** Classification of INFγ into six expression gradients in 716 CRCs. **(B)** IFNγ dosage dependent expression positive correlation (cc > 0.94) with six ICPs and three ICPRGs across six IFNγ expression level gradients (six CRC subsets).

To further define the dosage impact of IFNγ on the expression of six ICPs and three ICPRGs, we generated six IFNγ expression level gradients ^1^ IFNγ: FPKM > 5 (4 CRCs); (1) IFNγ: FPKM = 4.9–2 (20 CRCs); (2) IFNγ: FPKM = 1.99–1(44 CRCs); (3) IFNγ: FPKM = 0.99–0.5 (73 CRCs); (4) IFNγ: FPKM = 0.49–0.01 (467 CRC); and (5) IFNγ: FPKM < 0.009 (107 CRCs) in 716 CRCs (Indivumed [79 CRCs] and TCGA [637 CRCs]) (Figure 5A) and examined the impact of the levels on expression of the ICPs and ICPRGs examined in our more limited cohort. We found that the expression level of IFNγ was highly co-related (cc > 0.94) with expression levels of the six ICPs and three ICPRGs across the six IFNγ gradients (Figure 5B).

Regarding expression of ICPs and ICPRGs in matched normal control tissues, we found no differential expression of IFNγ, six ICPs and three ICPRGs between normal tissues adjacent to IFNγ positive vs. negative tumors (Tables S10, S11).

### Further Confirmation of IFNγ Dependent Expression of Six Classical ICPs and Three ICPRGs Among Five Other Solid Cancer Types

To evaluate whether our findings regarding IFNγ-associated expression of six classical ICPs and three ICPRGs in CRC also applied to distinct tumor types, we compared the overall stacked log_2_ expression of the six ICP genes and three ICPRGs in the following tumor types: 103 skin cutaneous melanomas (SKCMs); 1,105 breast cancers (BCs); 184 esophageal cancers (ESCs); 416 stomach cancers (STCs); and 501 lung squamous carcinomas (LUSC) all from the TCGA database. Similar to the CRC findings, the overall stacked log_2_ FPKM expression levels of six classical ICPs [CRC: (1.73-fold, *p* = 0.58), SKCM: (3.2-fold/*p* = 0.10), BC: (27.8-fold/*P* = 0.051), ESC: (2.1-fold/*p* = 0.39)], STC: (1.9-fold/ *p* = 0.51) and LUSC: (4.4-fold/ *p* = 0.10)] (Figure S4A) and three ICPRGs [CRC: (4.3-fold/ *p* = 0.29), SKCM: (5.4-fold/ *p* = 0.27), BC: (3.6-fold/ *p* = 0.33), ESC: (2.8-fold/ *p* = 0.36), STC: (3.1-fold/ *p* = 0.36) and LUSC: (2.9-fold/ *p* = 0.47)] (Figure S4B) were increased (1.7 to 27.8-fold) in IFNγ positive (FPKM > 1) tumors vs. IFNγ negative (FPKM < 1) tumors across these cancers but without statistical significance.

**Figure 6:**
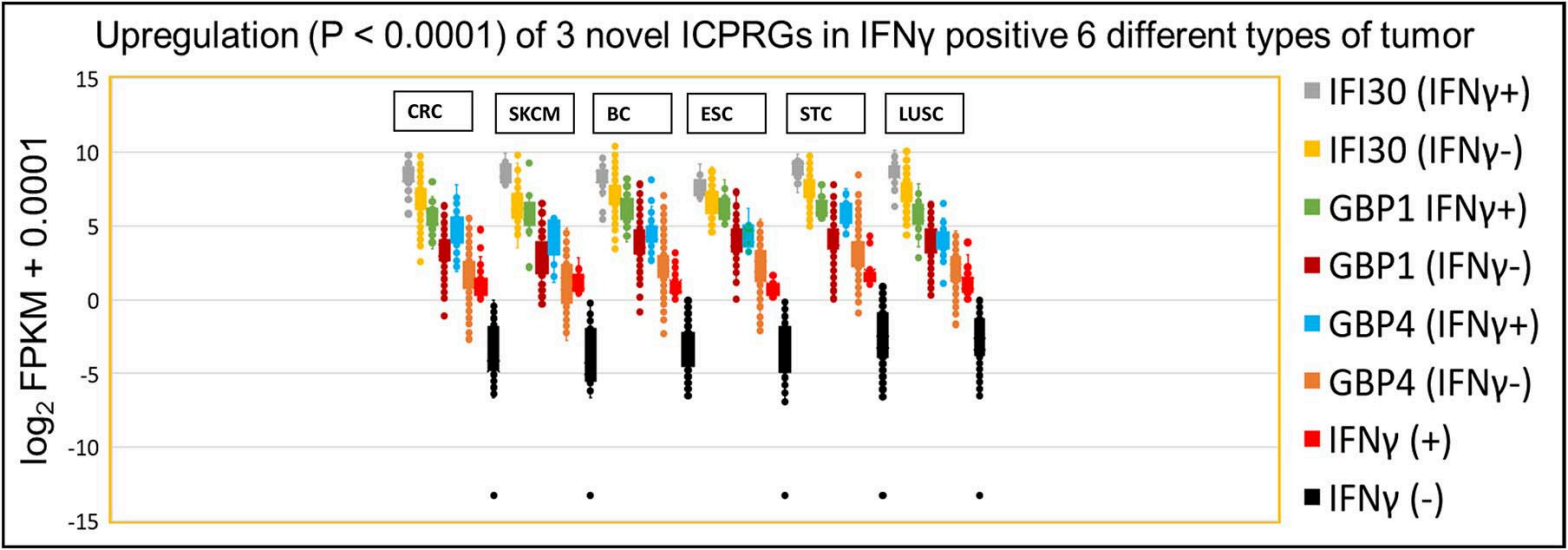
IFNγ dependent expression of three ICPRGs among five other solid cancers in Indivmed and TCGA cohort. Box and Whisker analysis of three ICPRGs in six types of cancer. Higher three ICPRGs and IFNγ expression in IFNγ (+) 69 CRCs (IFI30, 2.9-fold; GBP1, 4.6-fold; GBP4, 7.9-fold; and IFNγ, 44-fold), 13 SKCMs (IFI30, 4.6-fold; GBP1, 8.5-fold; GBP4, 8.3-fold; and IFNγ, 55-fold), 85 BCs (IFI30, 2.3-fold; GBP1, 4.9-fold; GBP4, 4.5-fold; and IFNγ, 19-fold), 13 ESCs (IFI30, 2.0-fold; GBP1, 4.5-fold; GBP4, 4.1-fold; and IFNγ 28-fold), 71 STCs (IFI30, 2.5-fold; GBP1, 4.5-fold; GBP4, 6.2-fold; and IFNγ 23-fold), and 68 LUSCs (IFI30, 2.6-fold; GBP1, 3.4-fold; GBP4, 4.1-fold; and IFNγ 17-fold), vs. IFNγ (–) 647 CRCs, 90 SKCMs, 1,020 BCs, 171 ESCs, 345 STCs, and 433 LUSCs.

Because these three ICPRGs have the potential to be novel actionable targets in cancer therapy, we further examined these individual genes by Box and Whisker plots of log_2_ FPKM expression levels in IFNγ positive and negative tumors of CRC, SKCM, BC, ESC, STC, and LUSC. IFI30, GBP1 and GBP4 were upregulated 2-8-fold (*p* < 0.0001) in IFNγ positive tumors vs. IFNγ negative tumors (Figure 6) in each of the six tumor types. Among these six cancers, only STC had a higher abundance (average FPKM) of IFI30, GBP1, and GBP4 (469, 78, 54) than did CRC (334, 47, 25) in the IFNγ positive tumors (Table S12). These three ICPRGs were also 24-fold more abundant than six ICPs in 5 types of normal tissues adjacent to BC, STC, LUSC, ESC, and CRC (Table S13) again raising the issue of whether these three genes are involved in a universal tolerance mechanism for normal tissues.

Strikingly in LUSC, all 68 IFNγ positive tumors had high expression of PD1 (FPKM > 1) while all 434 IFNγ negative tumors had low expression of PD1 (FPKM < 1) with a statistically significant 4.8-fold difference (Figure S5). These data suggest a dosage effect of IFNγ with respect to consequent expression of the six ICPs as well as for the three ICPRGs in six solid tumor types.

### Correlation of Immune Genes With Clinical Parameters in CRC

To address this issue, we separated by stage the 129 CRC pairs (79 CRC pairs as well as 50 CRC pairs/TCGA_38 cohort) into 57 low stage tumors (TNM stage I/II) and 82 high stage tumors (TNM stage III/IV) and found that there was no significant difference (*p* = 0.81) in the expression of IFI30, GBP1, GBP4, PD1, PDL1, CTLA4, Tim3, LAG3, IFNγ, and IDO1 between low and high stage CRCs (Figure S6). Because the tumor genetic profile may impact survival, we analyzed the Pathology Atlas data and found that (i) higher expression of IFI30, GBP1 and GBP4 was associated with better 5-year survival rate in breast cancer as well as in skin cutaneous melanoma, (ii) higher expression of GBP1 and GBP4 was associated with better 5-year survival rate in colorectal and stomach cancer, and (iii) higher expression of IFI30 was associated with worse 5-year survival rate in colorectal and stomach cancer (Table S14). These data suggest that any impact of IFI30, GBP1, and GBP4 on tumor response to diverse therapeutics is likely tumor type and context- dependent, clearly warranting further study.

## Discussion

Although IFNγ secreted by immune cells promotes growth arrest of tumors by augmenting MHC class I expression, contributing to the recruitment of effector cells, mediating Treg fragility and coordinating innate and adaptive antitumor responses (33, 34), IFNγ signaling can also compromise antitumor immunity by blocking these activities through the induction of immune checkpoint inhibitory molecules on T and tumor cells (35). The overall balance and timing of IFNγ expression over the course of tumor development and the downstream consequences likely critically determine an effective vs. suppressive immune response and an immunologic profile of consideration for immunotherapeutic approaches to treatment (36). In this study, we first demonstrated the specific upregulation and co-expression of six ICPs associated with higher expression of IFNγ in CRC. These data provide the molecular basis of using more than one ICP blocker in CRC with higher IFNγ expression but not lower IFNγ expression. Then, by analysis of genes co-expressed with IFNγ, we discovered three IFNγ associated ICPRGs. These three ICPRGs are expressed at higher abundance in CRC compared to the classical ICPs (except IDO1). Furthermore, there was differential co-expression of IFNγ with other immunologically pertinent genes between IFNγ positive and negative CRCs. IFNγ, the six ICPs, and the three ICPRGs were mainly co-expressed with T cell genes related to T cell activation, cytolysis and co-stimulation in IFNγ positive tumors while 2 ICPs and one ICPRG, but not IFNγ, were mainly co-expressed with B cell genes related to B cell activation, antigen presentation and response to lipopolysaccharide in IFNγ negative tumors. These data indicate dosage dependence of IFNγ on immune regulatory mechanisms in CRC. Finally, the co-upregulation of IFNγ with six ICPs as well as three ICPRGs was strongly supported by findings in the TCGA cohorts of melanoma, colon, breast, esophageal, stomach, and lung cancer. Thus, in addition to factors such as microsatellite stability status, tumor mutational burden, and expression of checkpoint inhibitory molecules, high IFNγ expression levels could potentially be investigated as a predictive biomarker for the potential for immune responsiveness of a tumor.

In our evaluation of the six classical ICPs, LAG3 appears to be a critical hub gene with the greatest number of co-expressed genes and though upregulated in IFNγ positive tumors, was downregulated and lacking co-expressed genes in IFNγ negative tumors. Thus, LAG3 may be a marker of biologically meaningful expression levels of IFNγ and an important drug target for CRC therapy in IFNγ positive CRC. The molecular mechanisms of LAG3 immune suppression have not been extensively defined. An additional ICP expressed at higher abundance compared to the other well-known ICP was IDO1, a rate-limiting metabolic enzyme that converts tryptophan into immune suppressive kynurenines (37). IDO1 is highly expressed in multiple types of human cancer (38) and studies indicate that while single-agent treatment with IDO1 enzyme inhibitor may not substantially decrease the established cancer burden, approaches combining select therapies with IDO1 blockade may have additive or synergistic effects, as shown in animal studies (39).

Based on their co-expression with six classical ICPs and with T cell markers, it is likely that the newly identified IFNγ related proteins, IFI30, GBP1, and GBP4 are immunomodulatory and may serve, in some tumors, as ICPs. That GBP1 and GBP4 are directly co-expressed with CD8A suggests the correlation of the three ICPRGs with a higher basal level of CD8A related infiltration in IFNγ positive CRC. It is well known that CD8^+^ T-cell infiltrates predict favorable prognosis in the majority of cancer types (40). In fact, GBP1 and GBP4 were associated with a favorable prognosis in 4 types of cancer (CRC, SKCM, BC, and STC) according to the Pathology Atlas analysis. These data are consistent with the evidence [KEYNOTE-001 trial/pembrolizumab (anti-PDL1) treatment] (41, 42) that high expression of PDL1, a classical immune suppressive check point molecule was associated with better survival among pembrolizumab-treated NSCLC and melanoma patients.

The immune and tumor related nature of these three genes are supported by the following published data: (i) IFI30 suppresses mouse primary T cell reactivity *in vitro* and mouse autoimmunity through cellular redox chemistry and ERK1/2 phosphorylation *in vivo*, promotes cell proliferation of a glioma cell line, but IFI30 RNA has been associated, with better patient survival rate in breast cancer and diffuse large B cell lymphoma (DLBCL) (9–14), (ii) GBP1 suppresses TCR signaling through lymphocyte cell-specific protein-tyrosine kinase and IL2 production in a human T cell line promotes cell proliferation/anti-apoptosis of a glioblastoma and two breast cancer cell lines, but inhibits cell proliferation of a colon cancer line. Furthermore, GBP1 reduces radioresistance of two human oral and liver cancer cell lines and correlates with better prognosis in melanoma but with poorer prognosis in human glioblastoma (15–21), (iii) GBP4 inhibits innate responses to viral infection (22) but lacks known tumor related functions to date. Thus, both knock-down and overexpression of these three genes should be tested in the future experiments to define the exact roles of these proteins within specific contexts. Additionally, there is the potential that inhibiting or stimulating them could change responses to infection and autoimmunity given their abundant expression in normal colonic tissues.

The co-expression of CTLA4 and PD1 with predominantly B cell markers and the co-expression of GBP1/GBP4 with six genes mainly related to anti-viral and microbial infection in normal intestinal epithelium [CXCL9, GBP5, STAT1, PARP9 (Poly ADP-Ribose Polymerase 9), TRAFD1 (Type Zinc Finger Domain Containing 1), and UBE2L6 (ubiquitin conjugating enzyme E2 L6)] suggest that maintenance of homeostasis, challenged by commensal bacteria, food antigens and potential autoantigens, may be maintained by B regulatory cell induction of ICPs (43–45). The relationship of these factors to mechanisms of intestinal tolerance and immunity clearly requires further study.

In summary, by applying NGS to study the expression of six classical ICPs and their co-expression networks, we found not only the well-established connection between IFNγ and the expression of ICPs in CRC, a relatively immunotherapy-refractory tumor type, but also, a novel set of ICPRGs as well as potential new hub genes which may be potential therapeutic targets. This study also provides comprehensive ICP co-expression information and fortifies the importance of NGS profiling in CRC and other tumors. The expression of higher abundance and novel ICPRG genes, including IFI30, GBP1, and GBP4, requires further evaluation of protein expression levels and immune inhibitory function in tumors.

## Data Availability Statement

The datasets generated for this study are available on request to the corresponding author.

## Author Contributions

RW, WW, J-NP, C-TL, and R-FS carried out experiments. RW, HM, L-HW, EP, W-LA, and LX performed data analysis. AR, LX, RW, LP, HM, EP, and HJ designed experiments and interpreted results. AR, RW, HM, LP, and LX wrote the manuscript, and all authors edited it. AR was the principal investigator of this study. All authors reviewed and approved the final manuscript.

### Conflict of Interest

HJ was employed by the company Indivumed GMBH. The remaining authors declare that the research was conducted in the absence of any commercial or financial relationships that could be construed as a potential conflict of interest.

## Footnotes

^1^https://www.cancer.org/cancer/colon-rectal-cancer/about/key-statistics.html
