# Supplementary material for: NGS Evaluation of Colorectal Cancer Reveals Interferon Gamma Dependent Expression of Immune Checkpoint Genes and Identification of Novel IFNγ Induced Genes
Source: Front Immunol. 2020 Mar 19;11:224. doi: 10.3389/fimmu.2020.00224 (PMC7103651; doi:10.3389/fimmu.2020.00224)
Supplement: Supplementary file 1 [file Presentation_1.pptx]

## Slide 1
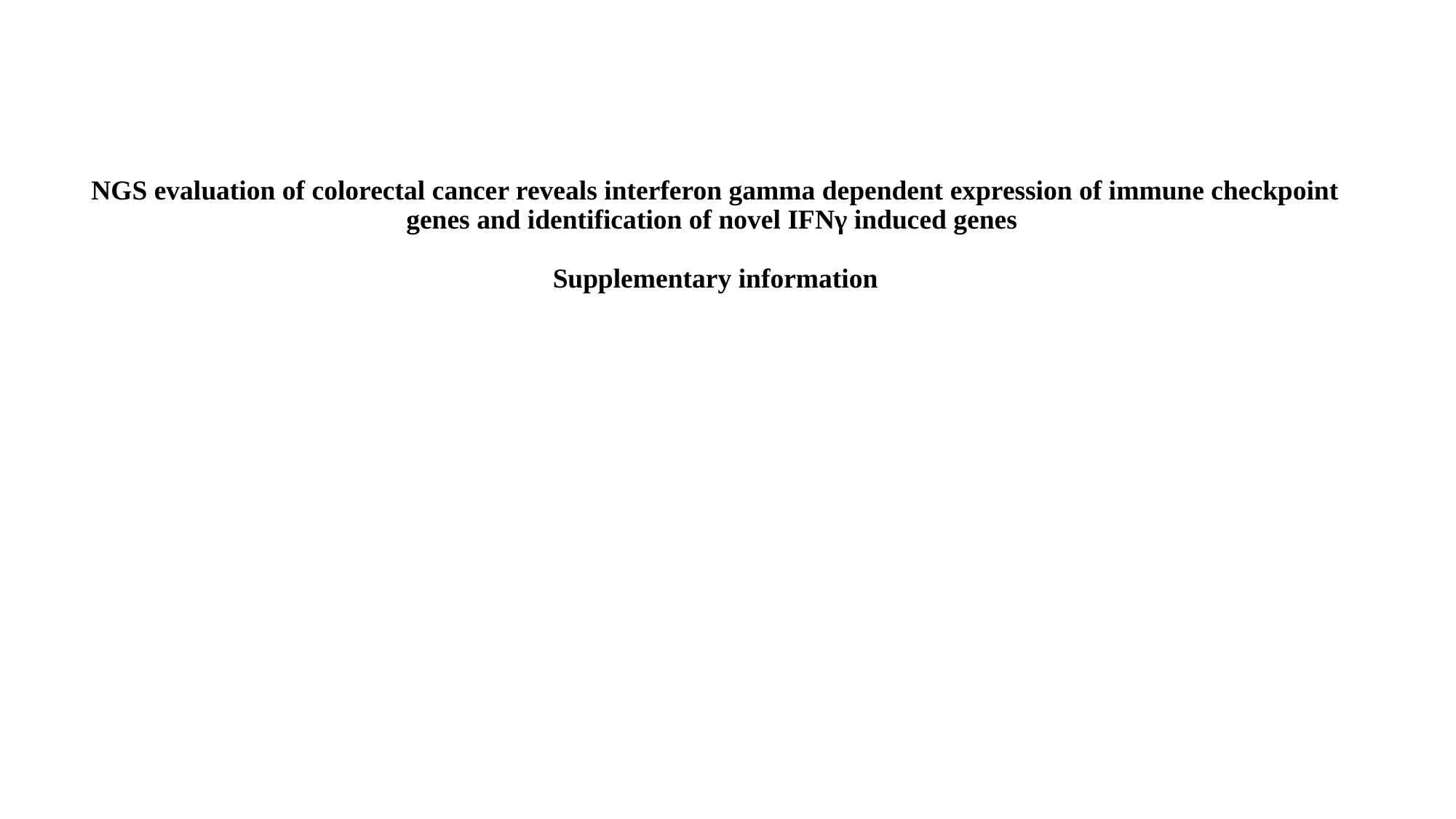

# NGS evaluation of colorectal cancer reveals interferon gamma dependent expression of immune checkpoint genes and identification of novel IFNγ induced genes Supplementary information

## Slide 2
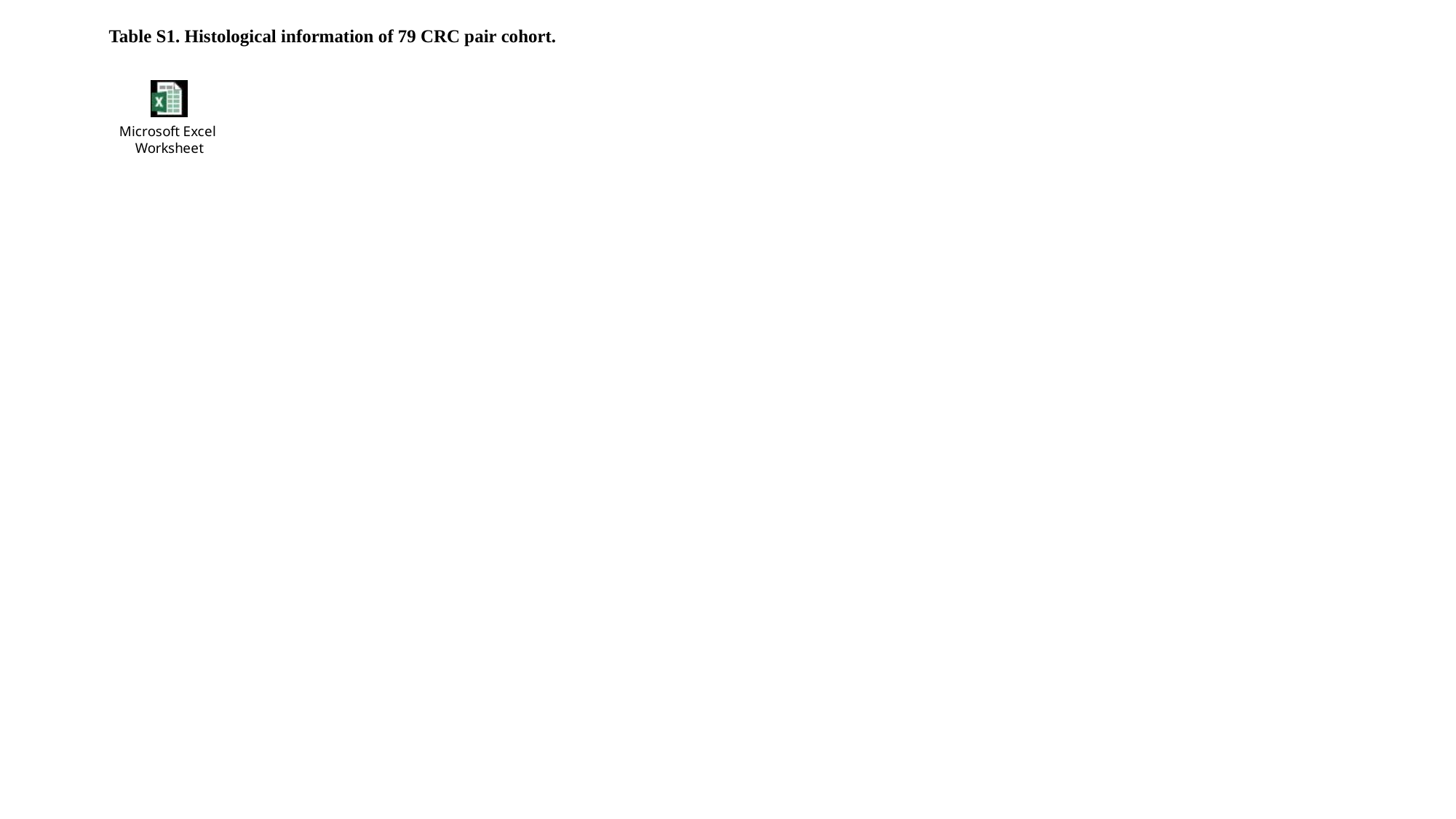

# Table S1. Histological information of 79 CRC pair cohort.

## Slide 3
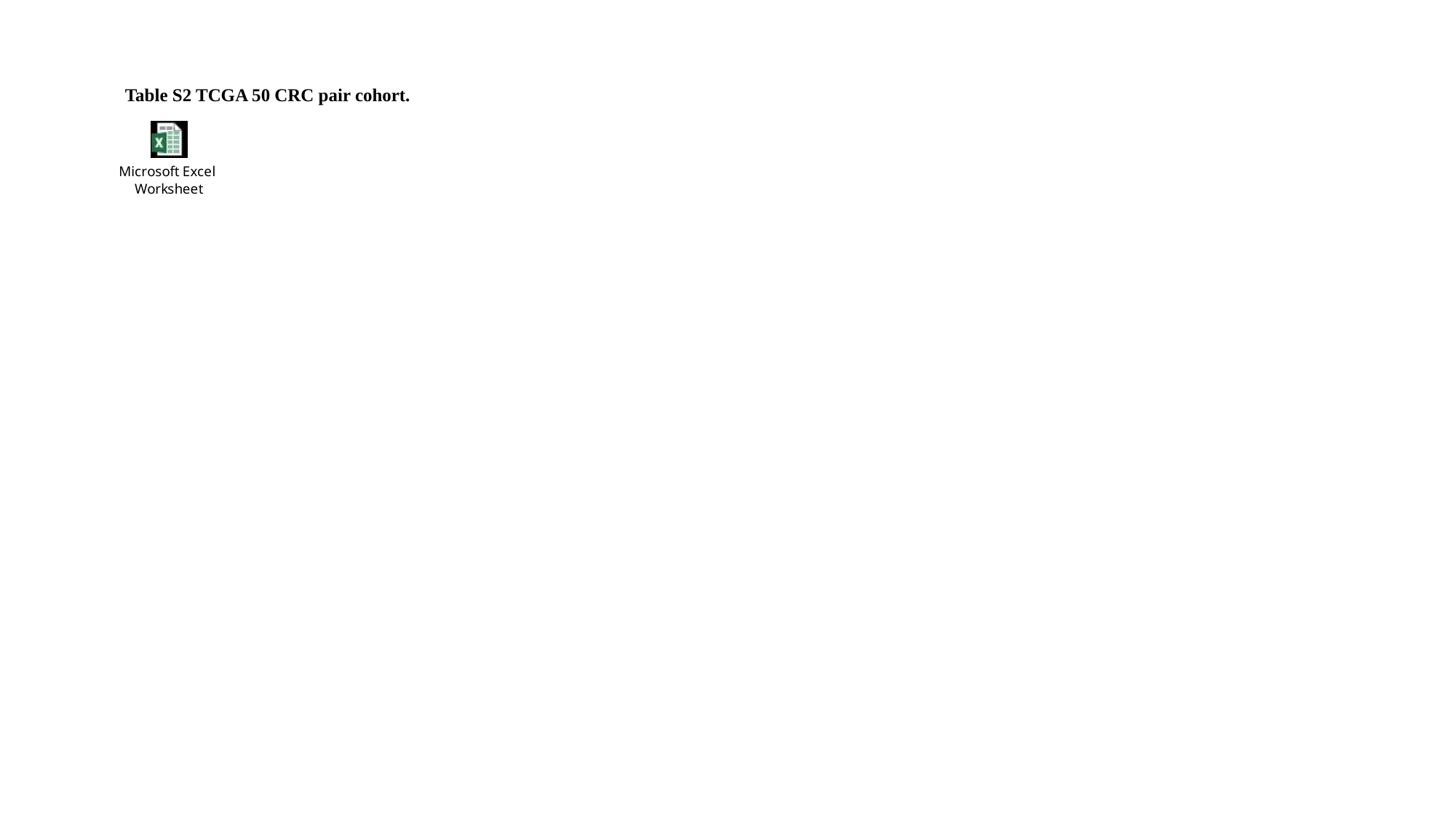

Table S2 TCGA 50 CRC pair cohort.

## Slide 4
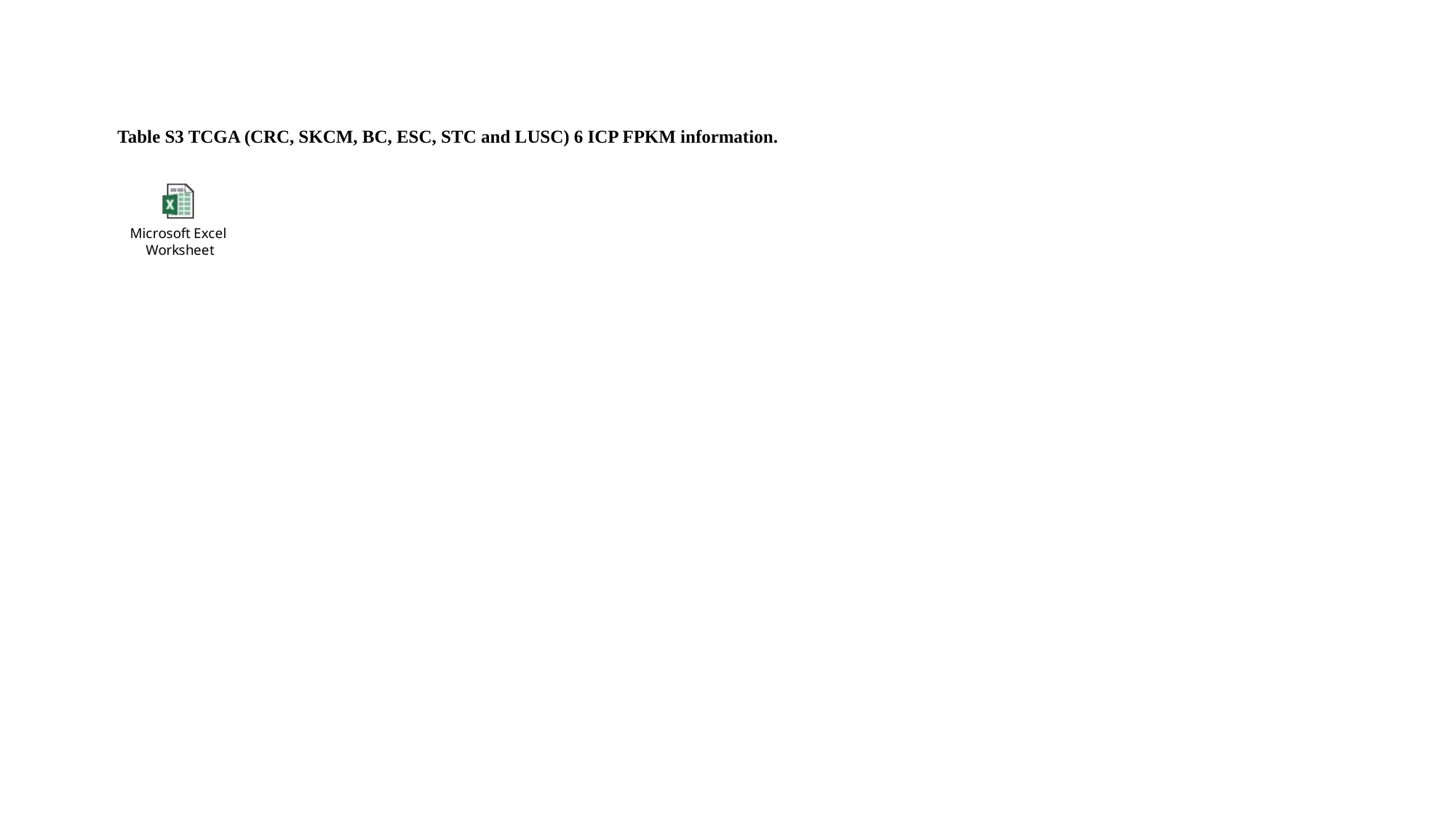

Table S3 TCGA (CRC, SKCM, BC, ESC, STC and LUSC) 6 ICP FPKM information.

## Slide 5
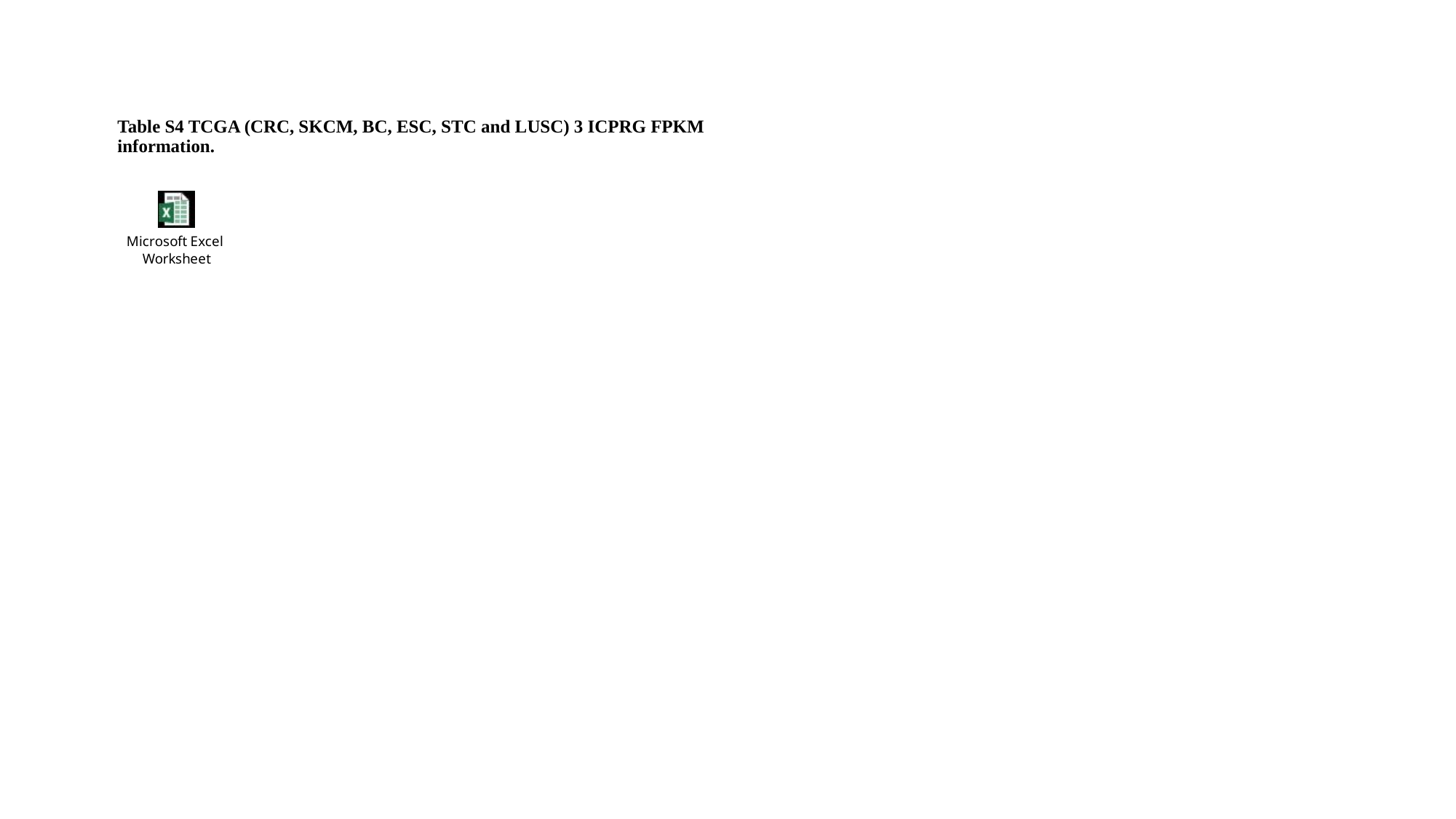

Table S4 TCGA (CRC, SKCM, BC, ESC, STC and LUSC) 3 ICPRG FPKM information.

## Slide 6
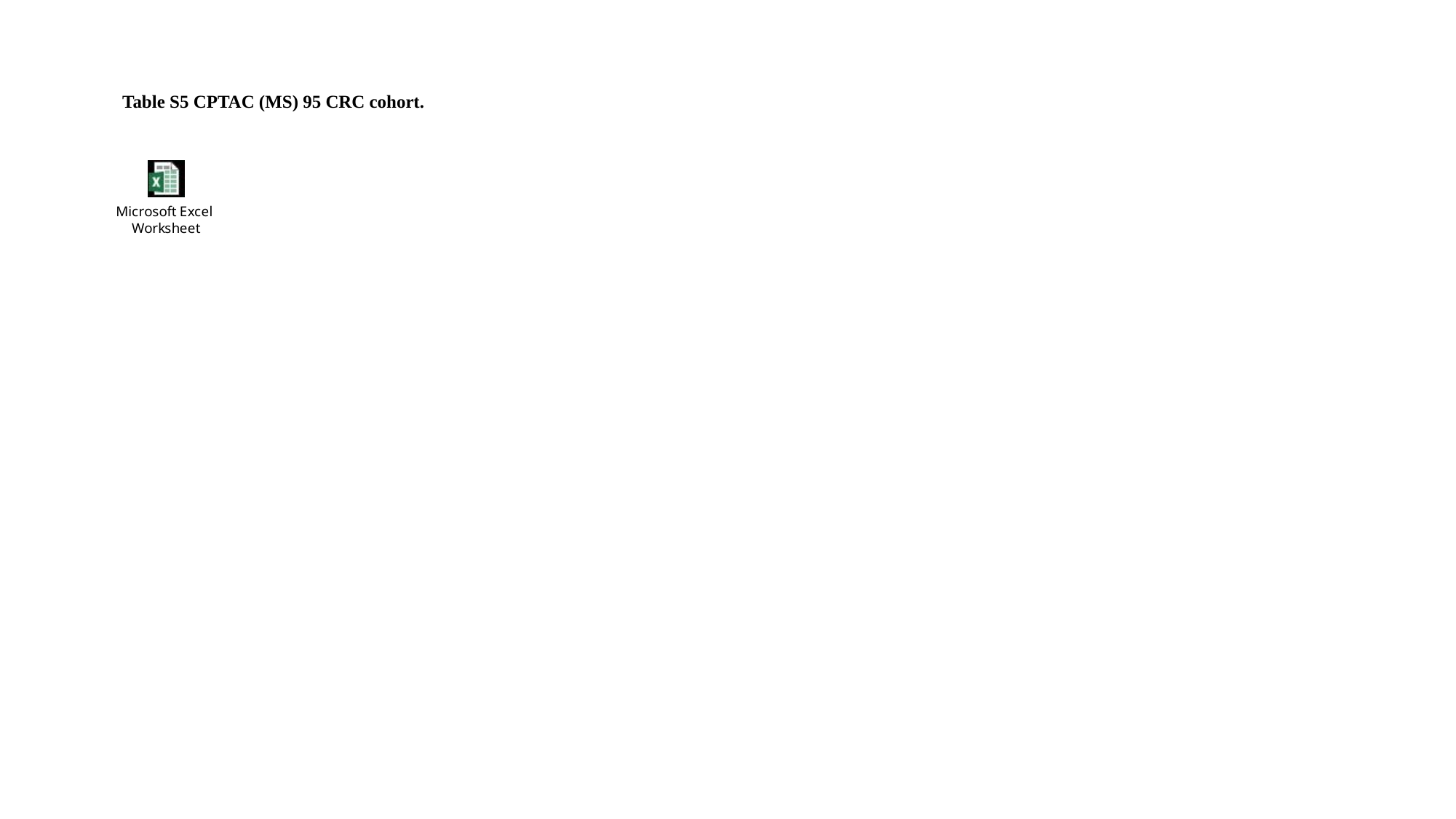

Table S5 CPTAC (MS) 95 CRC cohort.

## Slide 7
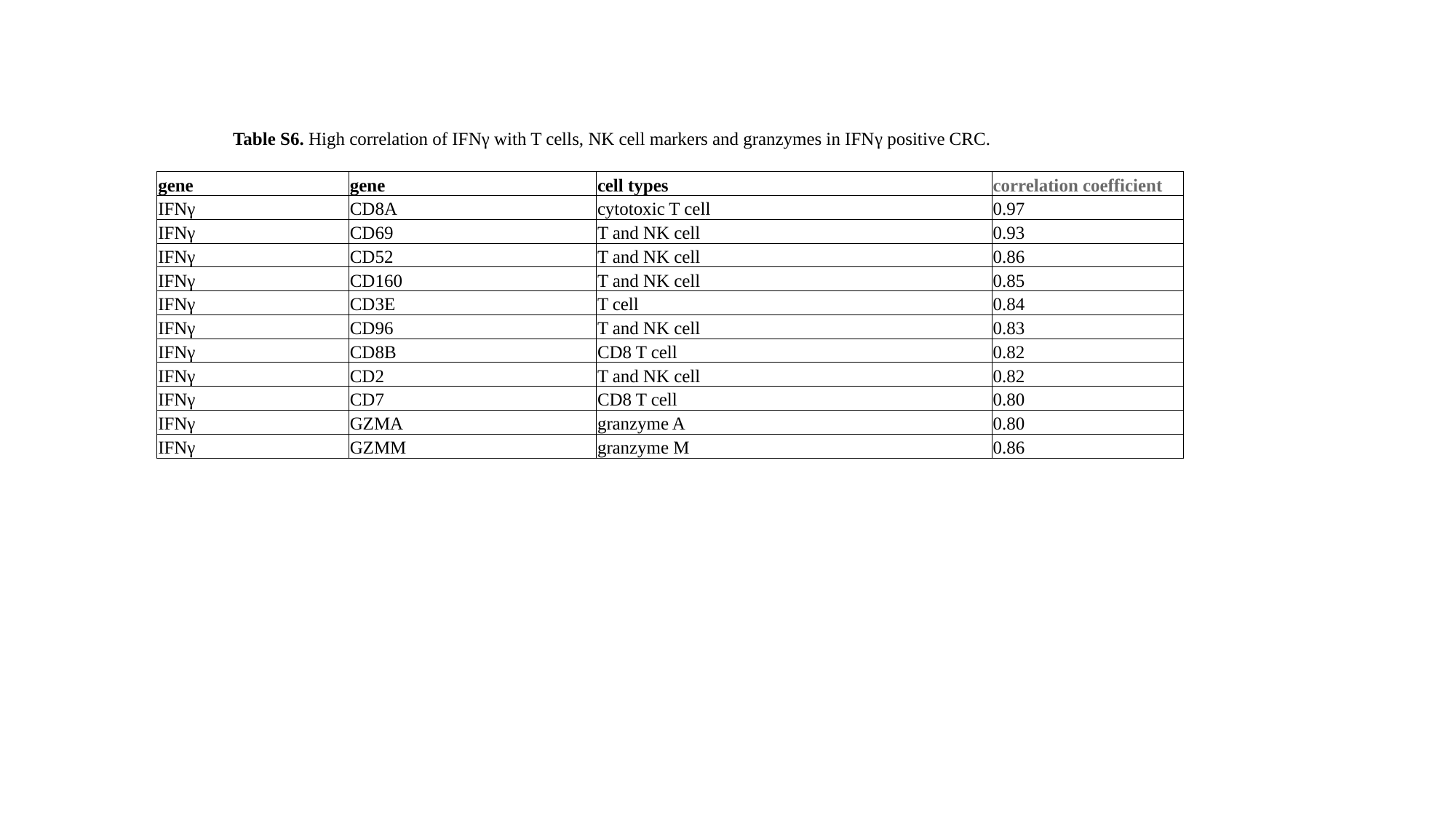

Table S6. High correlation of IFNγ with T cells, NK cell markers and granzymes in IFNγ positive CRC.
| gene | gene | cell types | correlation coefficient |
| --- | --- | --- | --- |
| IFNγ | CD8A | cytotoxic T cell | 0.97 |
| IFNγ | CD69 | T and NK cell | 0.93 |
| IFNγ | CD52 | T and NK cell | 0.86 |
| IFNγ | CD160 | T and NK cell | 0.85 |
| IFNγ | CD3E | T cell | 0.84 |
| IFNγ | CD96 | T and NK cell | 0.83 |
| IFNγ | CD8B | CD8 T cell | 0.82 |
| IFNγ | CD2 | T and NK cell | 0.82 |
| IFNγ | CD7 | CD8 T cell | 0.80 |
| IFNγ | GZMA | granzyme A | 0.80 |
| IFNγ | GZMM | granzyme M | 0.86 |

## Slide 8
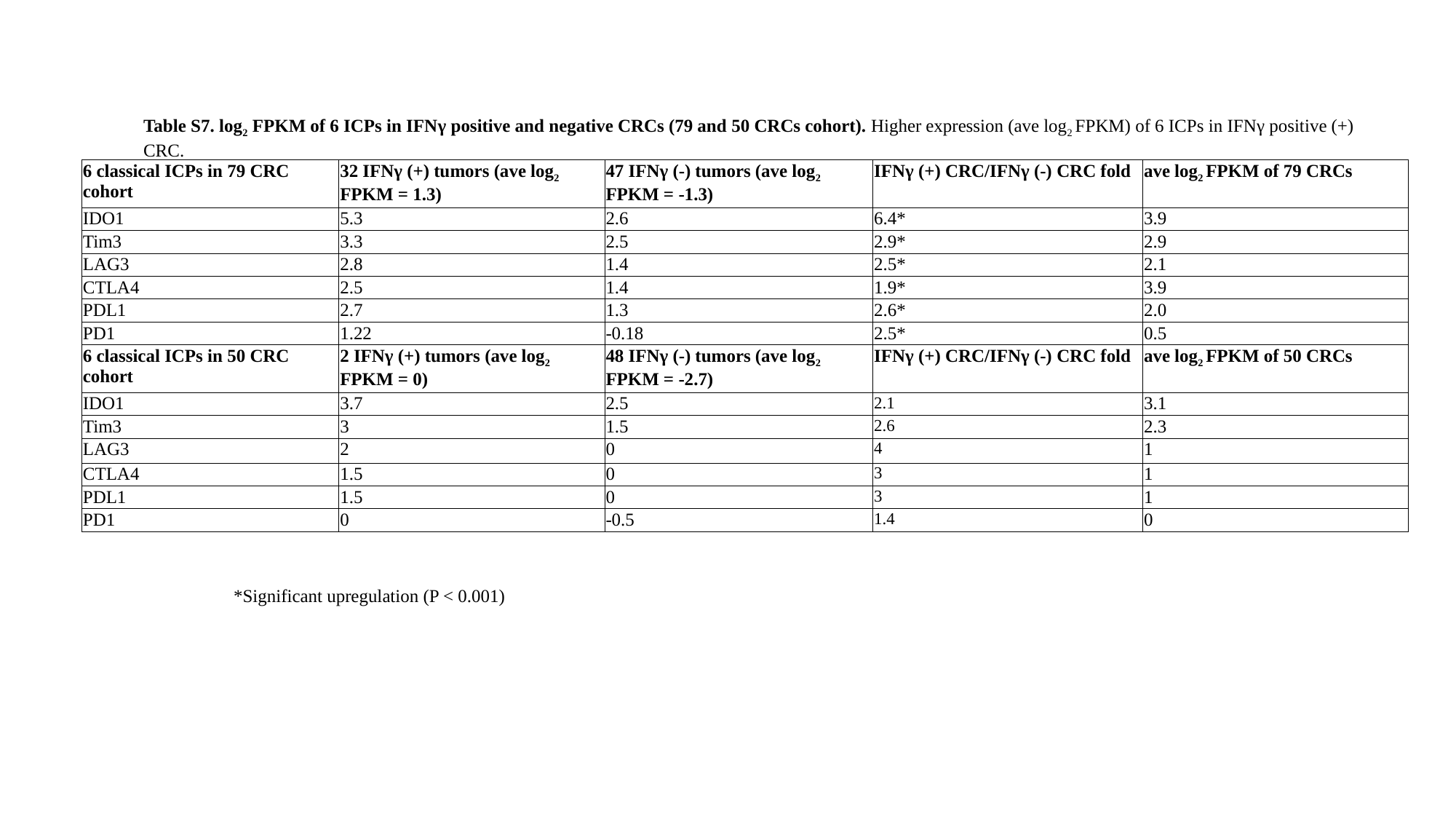

Table S7. log2 FPKM of 6 ICPs in IFNγ positive and negative CRCs (79 and 50 CRCs cohort). Higher expression (ave log2 FPKM) of 6 ICPs in IFNγ positive (+) CRC.
| 6 classical ICPs in 79 CRC cohort | 32 IFNγ (+) tumors (ave log2 FPKM = 1.3) | 47 IFNγ (-) tumors (ave log2 FPKM = -1.3) | IFNγ (+) CRC/IFNγ (-) CRC fold | ave log2 FPKM of 79 CRCs |
| --- | --- | --- | --- | --- |
| IDO1 | 5.3 | 2.6 | 6.4\* | 3.9 |
| Tim3 | 3.3 | 2.5 | 2.9\* | 2.9 |
| LAG3 | 2.8 | 1.4 | 2.5\* | 2.1 |
| CTLA4 | 2.5 | 1.4 | 1.9\* | 3.9 |
| PDL1 | 2.7 | 1.3 | 2.6\* | 2.0 |
| PD1 | 1.22 | -0.18 | 2.5\* | 0.5 |
| 6 classical ICPs in 50 CRC cohort | 2 IFNγ (+) tumors (ave log2 FPKM = 0) | 48 IFNγ (-) tumors (ave log2 FPKM = -2.7) | IFNγ (+) CRC/IFNγ (-) CRC fold | ave log2 FPKM of 50 CRCs |
| IDO1 | 3.7 | 2.5 | 2.1 | 3.1 |
| Tim3 | 3 | 1.5 | 2.6 | 2.3 |
| LAG3 | 2 | 0 | 4 | 1 |
| CTLA4 | 1.5 | 0 | 3 | 1 |
| PDL1 | 1.5 | 0 | 3 | 1 |
| PD1 | 0 | -0.5 | 1.4 | 0 |
*Significant upregulation (P < 0.001)

## Slide 9
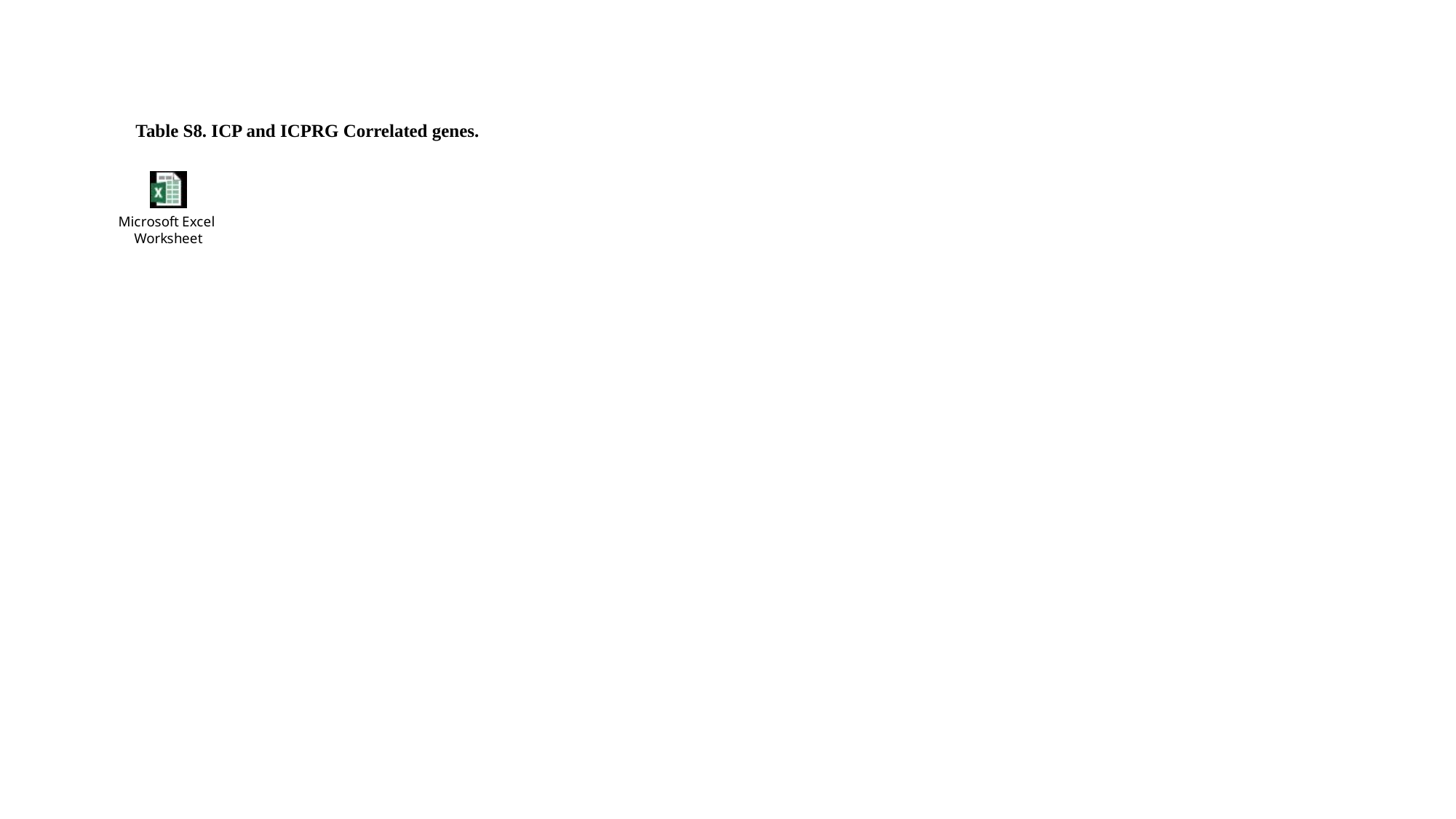

Table S8. ICP and ICPRG Correlated genes.

## Slide 10
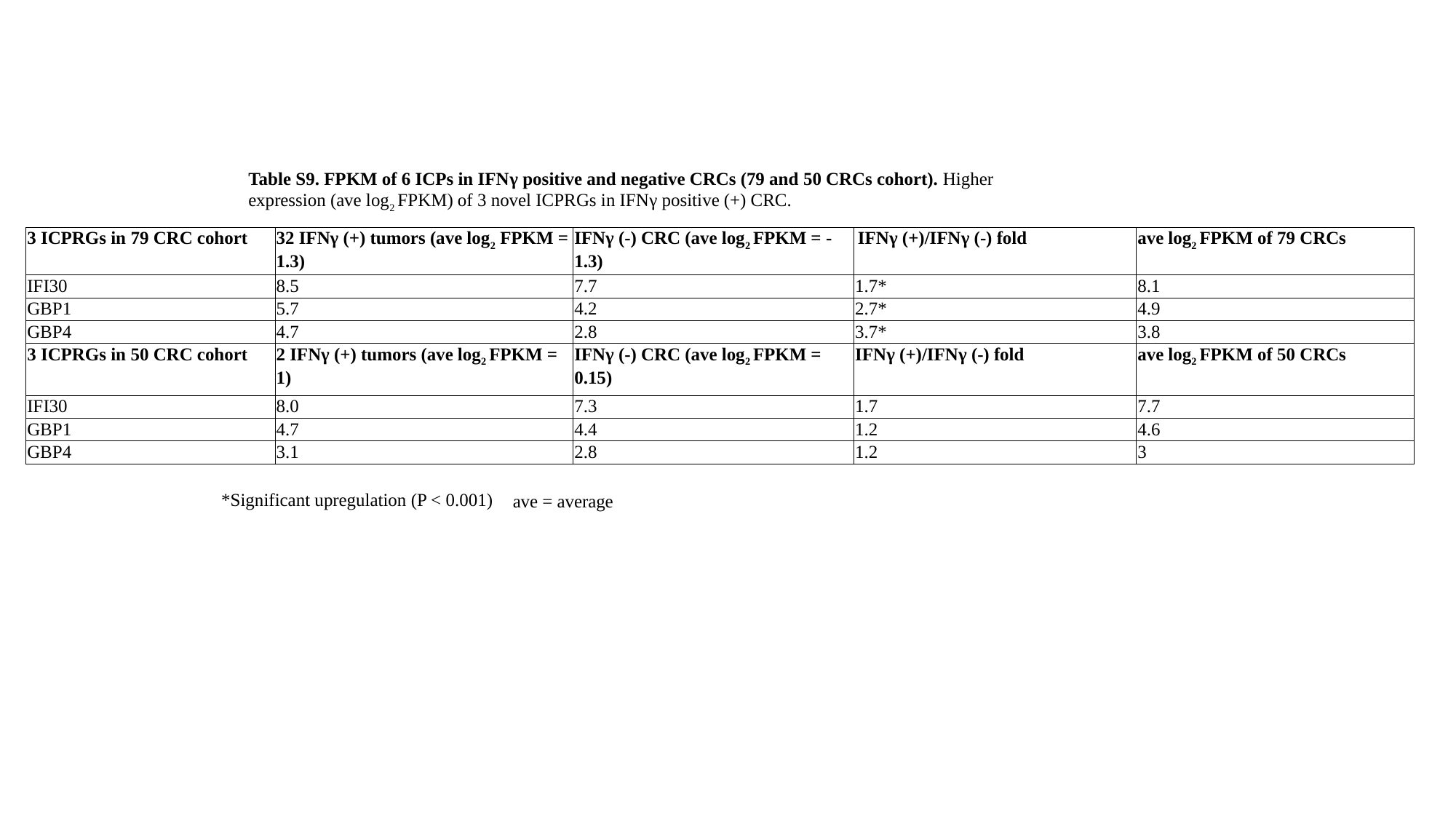

Table S9. FPKM of 6 ICPs in IFNγ positive and negative CRCs (79 and 50 CRCs cohort). Higher expression (ave log2 FPKM) of 3 novel ICPRGs in IFNγ positive (+) CRC.
| 3 ICPRGs in 79 CRC cohort | 32 IFNγ (+) tumors (ave log2 FPKM = 1.3) | IFNγ (-) CRC (ave log2 FPKM = -1.3) | IFNγ (+)/IFNγ (-) fold | ave log2 FPKM of 79 CRCs |
| --- | --- | --- | --- | --- |
| IFI30 | 8.5 | 7.7 | 1.7\* | 8.1 |
| GBP1 | 5.7 | 4.2 | 2.7\* | 4.9 |
| GBP4 | 4.7 | 2.8 | 3.7\* | 3.8 |
| 3 ICPRGs in 50 CRC cohort | 2 IFNγ (+) tumors (ave log2 FPKM = 1) | IFNγ (-) CRC (ave log2 FPKM = 0.15) | IFNγ (+)/IFNγ (-) fold | ave log2 FPKM of 50 CRCs |
| IFI30 | 8.0 | 7.3 | 1.7 | 7.7 |
| GBP1 | 4.7 | 4.4 | 1.2 | 4.6 |
| GBP4 | 3.1 | 2.8 | 1.2 | 3 |
*Significant upregulation (P < 0.001)
ave = average

## Slide 11
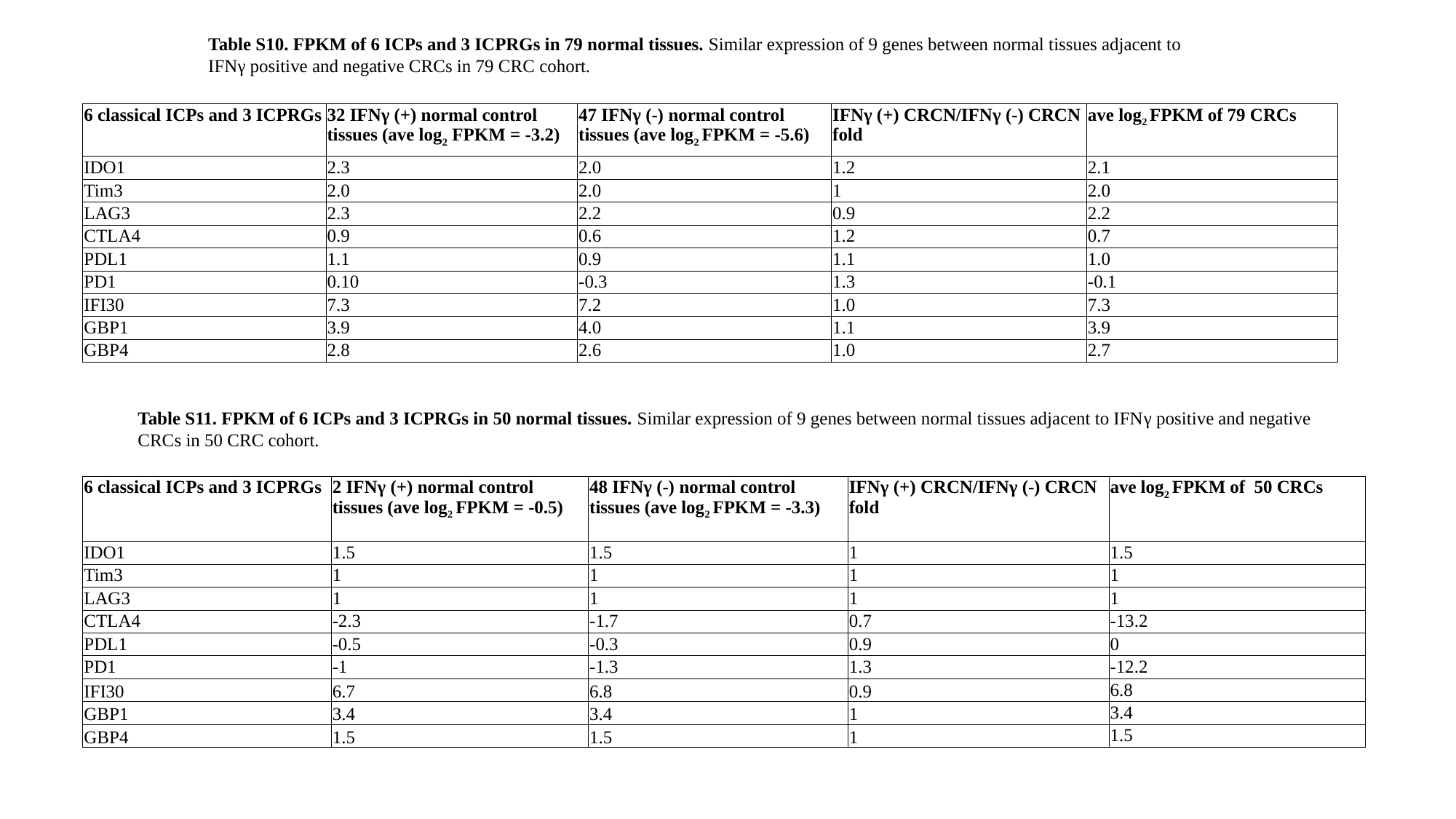

Table S10. FPKM of 6 ICPs and 3 ICPRGs in 79 normal tissues. Similar expression of 9 genes between normal tissues adjacent to IFNγ positive and negative CRCs in 79 CRC cohort.
| 6 classical ICPs and 3 ICPRGs | 32 IFNγ (+) normal control tissues (ave log2 FPKM = -3.2) | 47 IFNγ (-) normal control tissues (ave log2 FPKM = -5.6) | IFNγ (+) CRCN/IFNγ (-) CRCN fold | ave log2 FPKM of 79 CRCs |
| --- | --- | --- | --- | --- |
| IDO1 | 2.3 | 2.0 | 1.2 | 2.1 |
| Tim3 | 2.0 | 2.0 | 1 | 2.0 |
| LAG3 | 2.3 | 2.2 | 0.9 | 2.2 |
| CTLA4 | 0.9 | 0.6 | 1.2 | 0.7 |
| PDL1 | 1.1 | 0.9 | 1.1 | 1.0 |
| PD1 | 0.10 | -0.3 | 1.3 | -0.1 |
| IFI30 | 7.3 | 7.2 | 1.0 | 7.3 |
| GBP1 | 3.9 | 4.0 | 1.1 | 3.9 |
| GBP4 | 2.8 | 2.6 | 1.0 | 2.7 |
Table S11. FPKM of 6 ICPs and 3 ICPRGs in 50 normal tissues. Similar expression of 9 genes between normal tissues adjacent to IFNγ positive and negative CRCs in 50 CRC cohort.
| 6 classical ICPs and 3 ICPRGs | 2 IFNγ (+) normal control tissues (ave log2 FPKM = -0.5) | 48 IFNγ (-) normal control tissues (ave log2 FPKM = -3.3) | IFNγ (+) CRCN/IFNγ (-) CRCN fold | ave log2 FPKM of 50 CRCs |
| --- | --- | --- | --- | --- |
| IDO1 | 1.5 | 1.5 | 1 | 1.5 |
| Tim3 | 1 | 1 | 1 | 1 |
| LAG3 | 1 | 1 | 1 | 1 |
| CTLA4 | -2.3 | -1.7 | 0.7 | -13.2 |
| PDL1 | -0.5 | -0.3 | 0.9 | 0 |
| PD1 | -1 | -1.3 | 1.3 | -12.2 |
| IFI30 | 6.7 | 6.8 | 0.9 | 6.8 |
| GBP1 | 3.4 | 3.4 | 1 | 3.4 |
| GBP4 | 1.5 | 1.5 | 1 | 1.5 |

## Slide 12
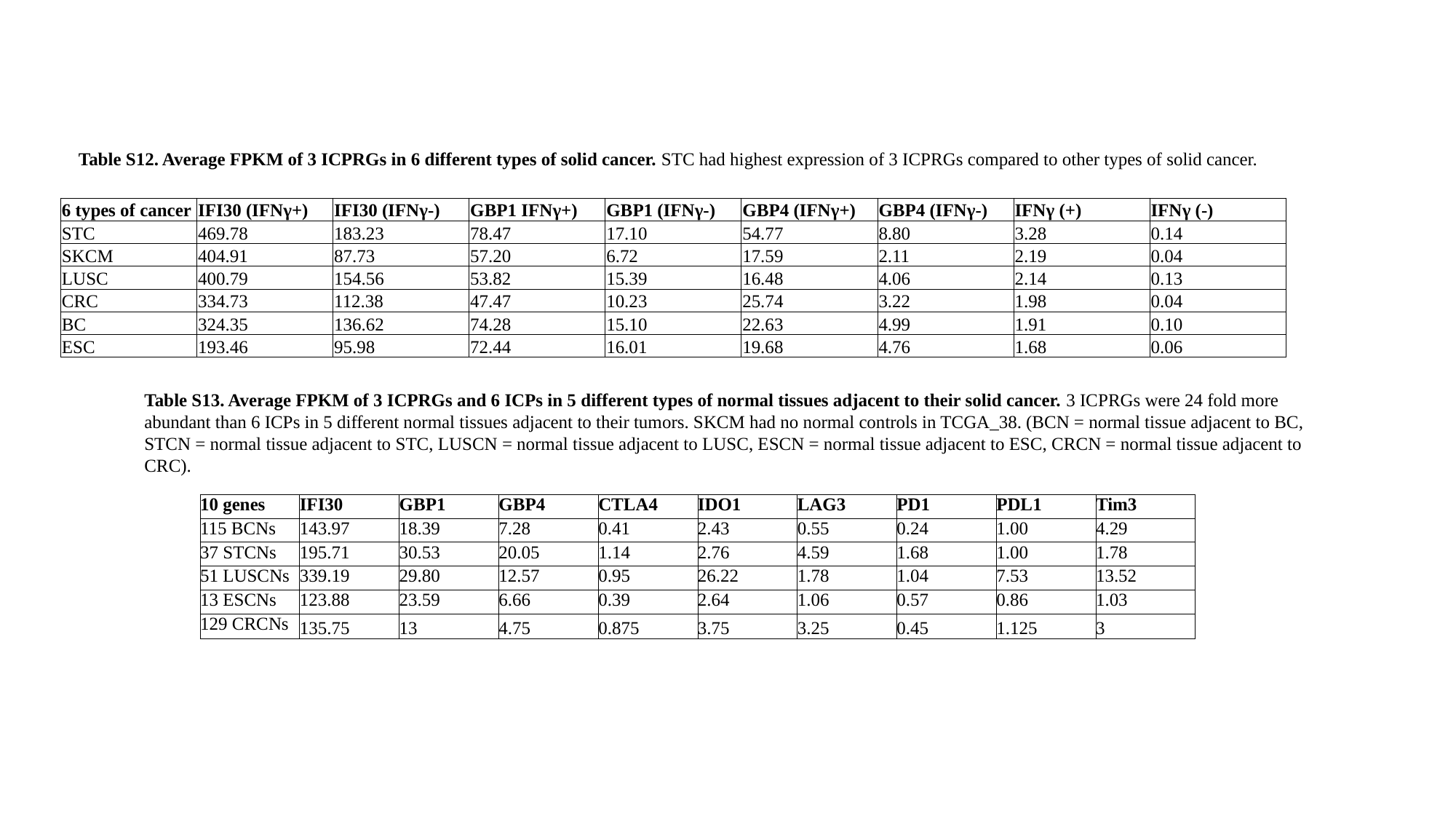

Table S12. Average FPKM of 3 ICPRGs in 6 different types of solid cancer. STC had highest expression of 3 ICPRGs compared to other types of solid cancer.
| 6 types of cancer | IFI30 (IFNγ+) | IFI30 (IFNγ-) | GBP1 IFNγ+) | GBP1 (IFNγ-) | GBP4 (IFNγ+) | GBP4 (IFNγ-) | IFNγ (+) | IFNγ (-) |
| --- | --- | --- | --- | --- | --- | --- | --- | --- |
| STC | 469.78 | 183.23 | 78.47 | 17.10 | 54.77 | 8.80 | 3.28 | 0.14 |
| SKCM | 404.91 | 87.73 | 57.20 | 6.72 | 17.59 | 2.11 | 2.19 | 0.04 |
| LUSC | 400.79 | 154.56 | 53.82 | 15.39 | 16.48 | 4.06 | 2.14 | 0.13 |
| CRC | 334.73 | 112.38 | 47.47 | 10.23 | 25.74 | 3.22 | 1.98 | 0.04 |
| BC | 324.35 | 136.62 | 74.28 | 15.10 | 22.63 | 4.99 | 1.91 | 0.10 |
| ESC | 193.46 | 95.98 | 72.44 | 16.01 | 19.68 | 4.76 | 1.68 | 0.06 |
Table S13. Average FPKM of 3 ICPRGs and 6 ICPs in 5 different types of normal tissues adjacent to their solid cancer. 3 ICPRGs were 24 fold more abundant than 6 ICPs in 5 different normal tissues adjacent to their tumors. SKCM had no normal controls in TCGA_38. (BCN = normal tissue adjacent to BC, STCN = normal tissue adjacent to STC, LUSCN = normal tissue adjacent to LUSC, ESCN = normal tissue adjacent to ESC, CRCN = normal tissue adjacent to CRC).
| 10 genes | IFI30 | GBP1 | GBP4 | CTLA4 | IDO1 | LAG3 | PD1 | PDL1 | Tim3 |
| --- | --- | --- | --- | --- | --- | --- | --- | --- | --- |
| 115 BCNs | 143.97 | 18.39 | 7.28 | 0.41 | 2.43 | 0.55 | 0.24 | 1.00 | 4.29 |
| 37 STCNs | 195.71 | 30.53 | 20.05 | 1.14 | 2.76 | 4.59 | 1.68 | 1.00 | 1.78 |
| 51 LUSCNs | 339.19 | 29.80 | 12.57 | 0.95 | 26.22 | 1.78 | 1.04 | 7.53 | 13.52 |
| 13 ESCNs | 123.88 | 23.59 | 6.66 | 0.39 | 2.64 | 1.06 | 0.57 | 0.86 | 1.03 |
| 129 CRCNs | 135.75 | 13 | 4.75 | 0.875 | 3.75 | 3.25 | 0.45 | 1.125 | 3 |

## Slide 13
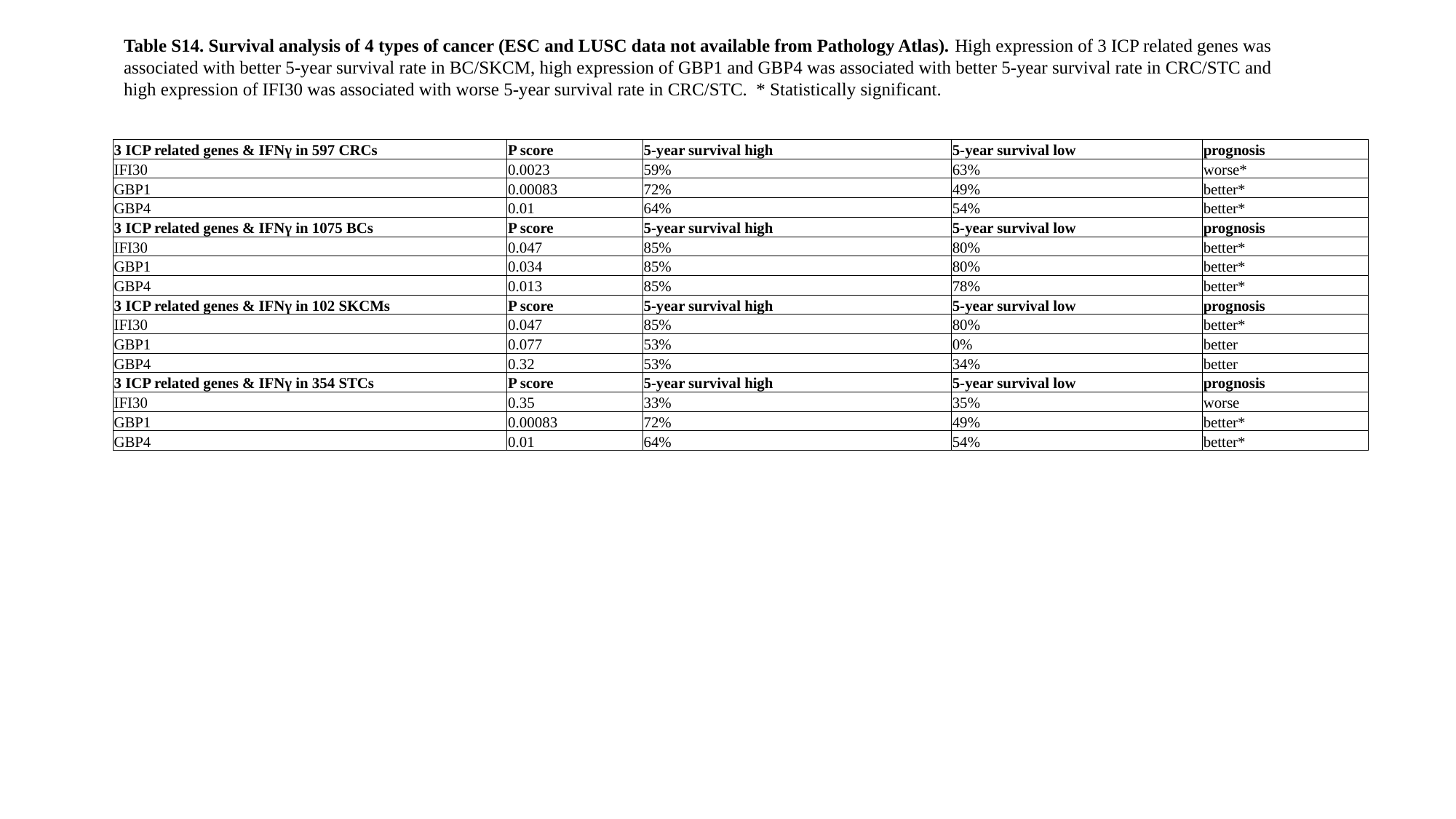

Table S14. Survival analysis of 4 types of cancer (ESC and LUSC data not available from Pathology Atlas). High expression of 3 ICP related genes was associated with better 5-year survival rate in BC/SKCM, high expression of GBP1 and GBP4 was associated with better 5-year survival rate in CRC/STC and high expression of IFI30 was associated with worse 5-year survival rate in CRC/STC. * Statistically significant.
| 3 ICP related genes & IFNγ in 597 CRCs | P score | 5-year survival high | 5-year survival low | prognosis |
| --- | --- | --- | --- | --- |
| IFI30 | 0.0023 | 59% | 63% | worse\* |
| GBP1 | 0.00083 | 72% | 49% | better\* |
| GBP4 | 0.01 | 64% | 54% | better\* |
| 3 ICP related genes & IFNγ in 1075 BCs | P score | 5-year survival high | 5-year survival low | prognosis |
| IFI30 | 0.047 | 85% | 80% | better\* |
| GBP1 | 0.034 | 85% | 80% | better\* |
| GBP4 | 0.013 | 85% | 78% | better\* |
| 3 ICP related genes & IFNγ in 102 SKCMs | P score | 5-year survival high | 5-year survival low | prognosis |
| IFI30 | 0.047 | 85% | 80% | better\* |
| GBP1 | 0.077 | 53% | 0% | better |
| GBP4 | 0.32 | 53% | 34% | better |
| 3 ICP related genes & IFNγ in 354 STCs | P score | 5-year survival high | 5-year survival low | prognosis |
| IFI30 | 0.35 | 33% | 35% | worse |
| GBP1 | 0.00083 | 72% | 49% | better\* |
| GBP4 | 0.01 | 64% | 54% | better\* |

## Slide 14
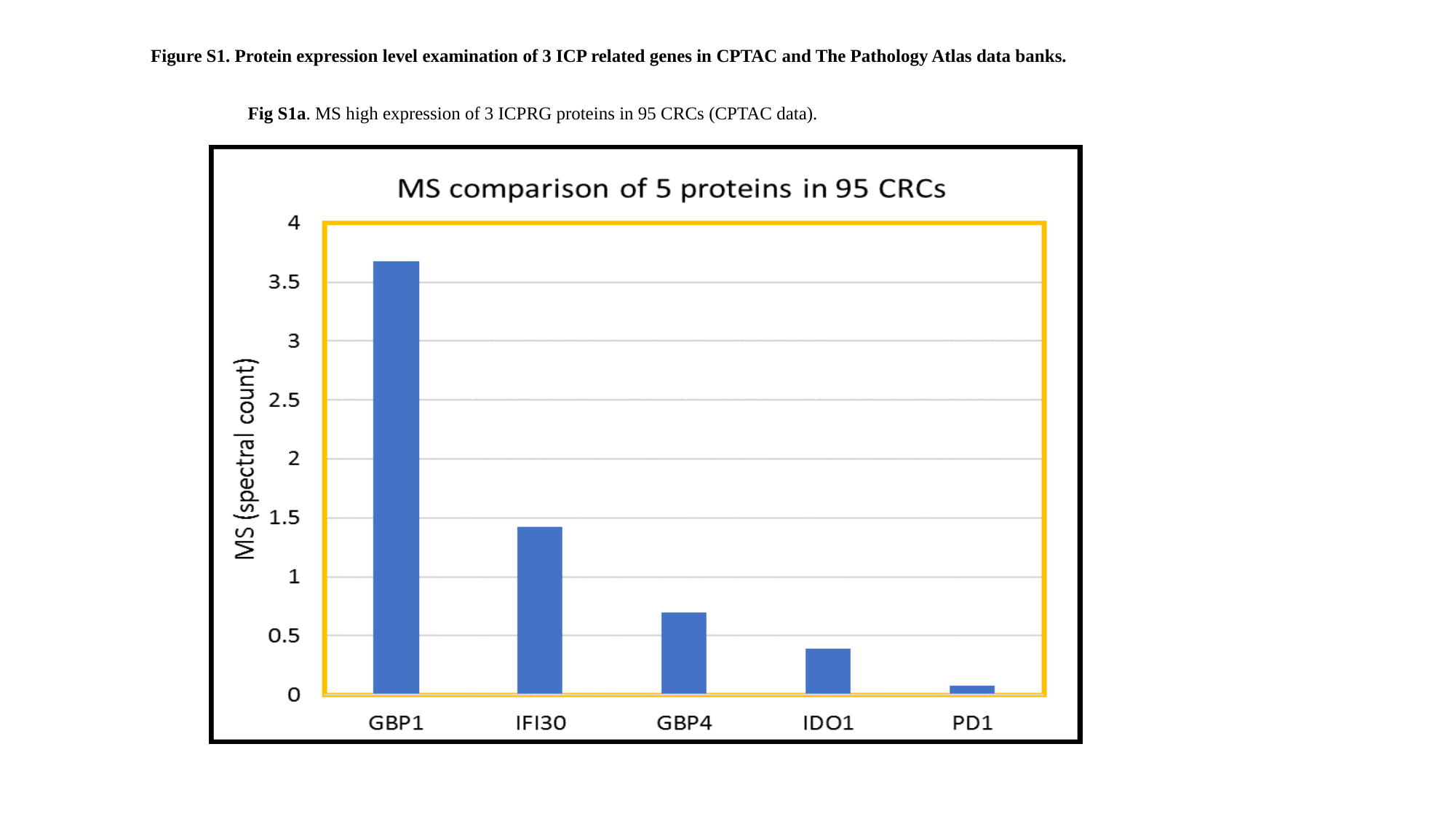

Figure S1. Protein expression level examination of 3 ICP related genes in CPTAC and The Pathology Atlas data banks.
Fig S1a. MS high expression of 3 ICPRG proteins in 95 CRCs (CPTAC data).

## Slide 15
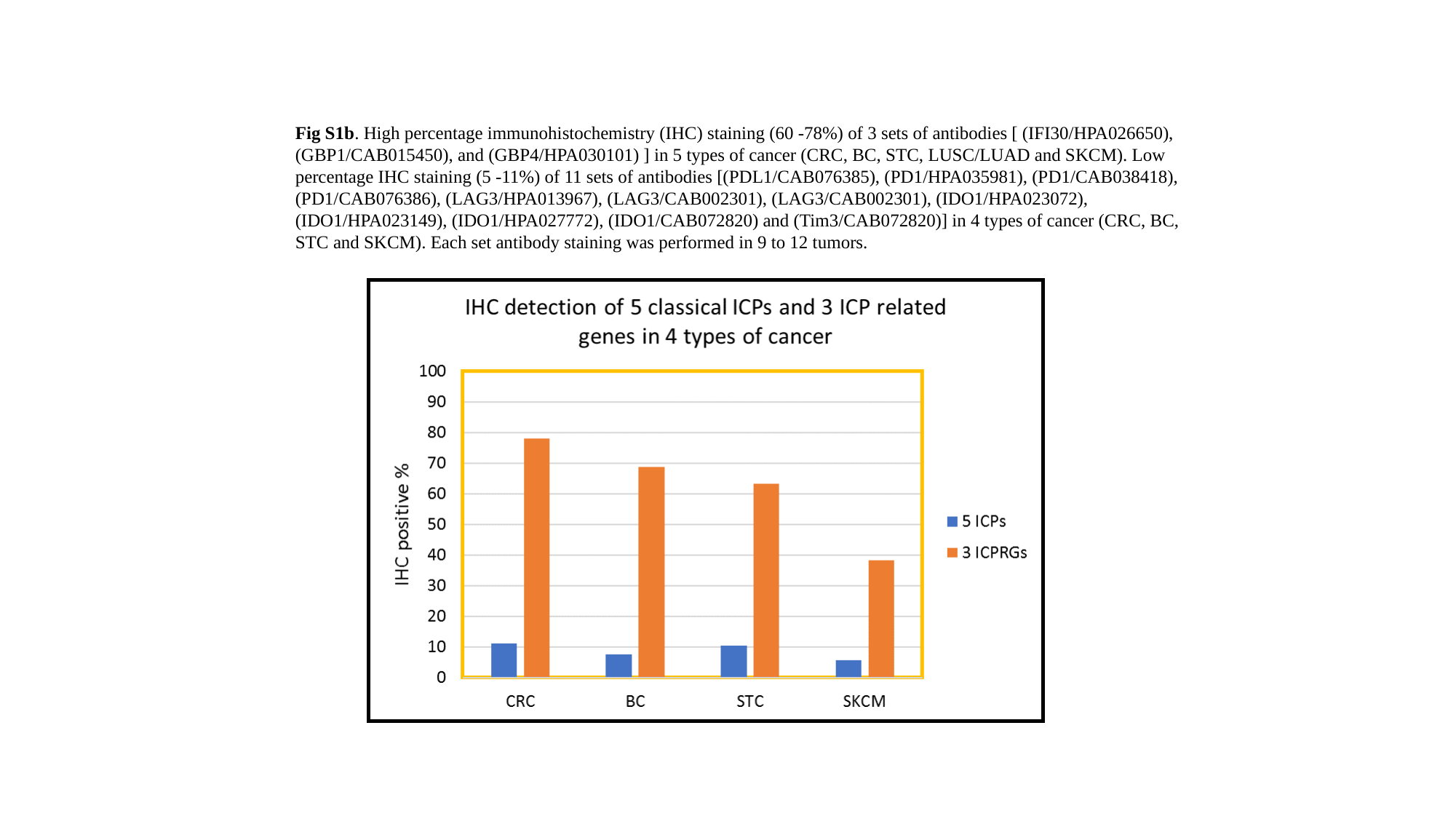

Fig S1b. High percentage immunohistochemistry (IHC) staining (60 -78%) of 3 sets of antibodies [ (IFI30/HPA026650), (GBP1/CAB015450), and (GBP4/HPA030101) ] in 5 types of cancer (CRC, BC, STC, LUSC/LUAD and SKCM). Low percentage IHC staining (5 -11%) of 11 sets of antibodies [(PDL1/CAB076385), (PD1/HPA035981), (PD1/CAB038418), (PD1/CAB076386), (LAG3/HPA013967), (LAG3/CAB002301), (LAG3/CAB002301), (IDO1/HPA023072), (IDO1/HPA023149), (IDO1/HPA027772), (IDO1/CAB072820) and (Tim3/CAB072820)] in 4 types of cancer (CRC, BC, STC and SKCM). Each set antibody staining was performed in 9 to 12 tumors.

## Slide 16
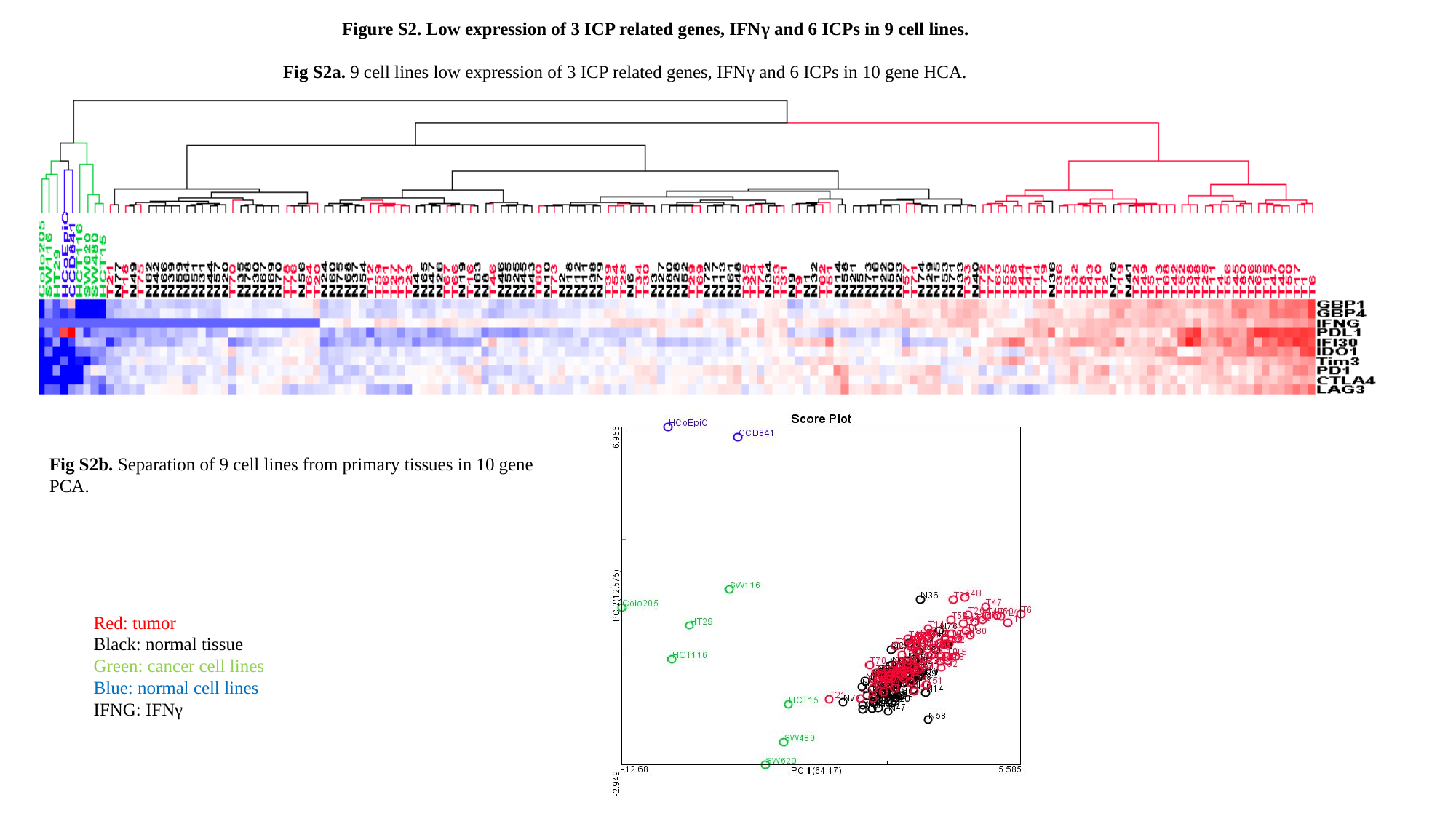

Figure S2. Low expression of 3 ICP related genes, IFNγ and 6 ICPs in 9 cell lines.
Fig S2a. 9 cell lines low expression of 3 ICP related genes, IFNγ and 6 ICPs in 10 gene HCA.
Fig S2b. Separation of 9 cell lines from primary tissues in 10 gene PCA.
Red: tumor
Black: normal tissue
Green: cancer cell lines
Blue: normal cell lines
IFNG: IFNγ

## Slide 17
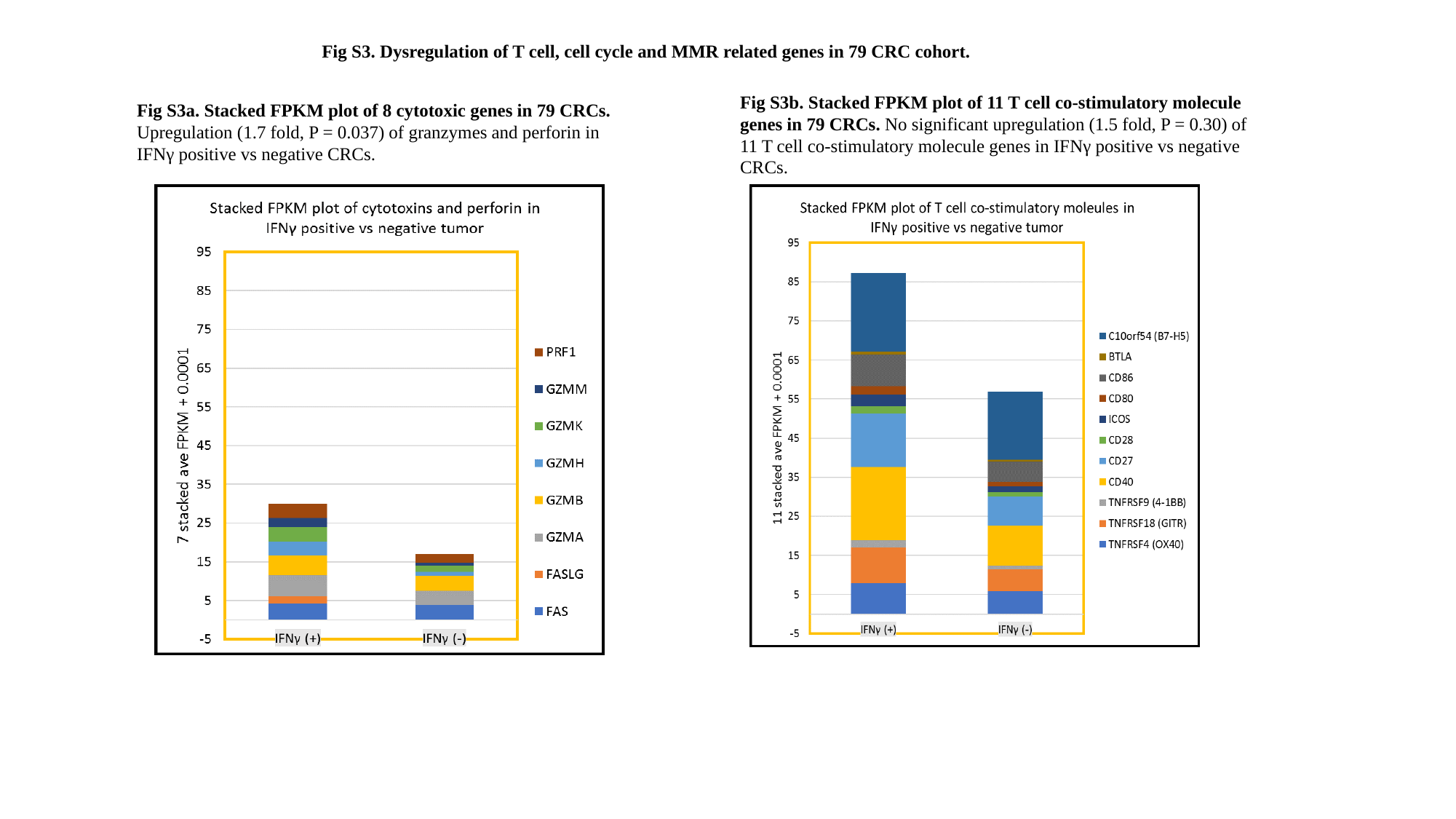

Fig S3. Dysregulation of T cell, cell cycle and MMR related genes in 79 CRC cohort.
Fig S3b. Stacked FPKM plot of 11 T cell co-stimulatory molecule genes in 79 CRCs. No significant upregulation (1.5 fold, P = 0.30) of 11 T cell co-stimulatory molecule genes in IFNγ positive vs negative CRCs.
Fig S3a. Stacked FPKM plot of 8 cytotoxic genes in 79 CRCs. Upregulation (1.7 fold, P = 0.037) of granzymes and perforin in IFNγ positive vs negative CRCs.

## Slide 18
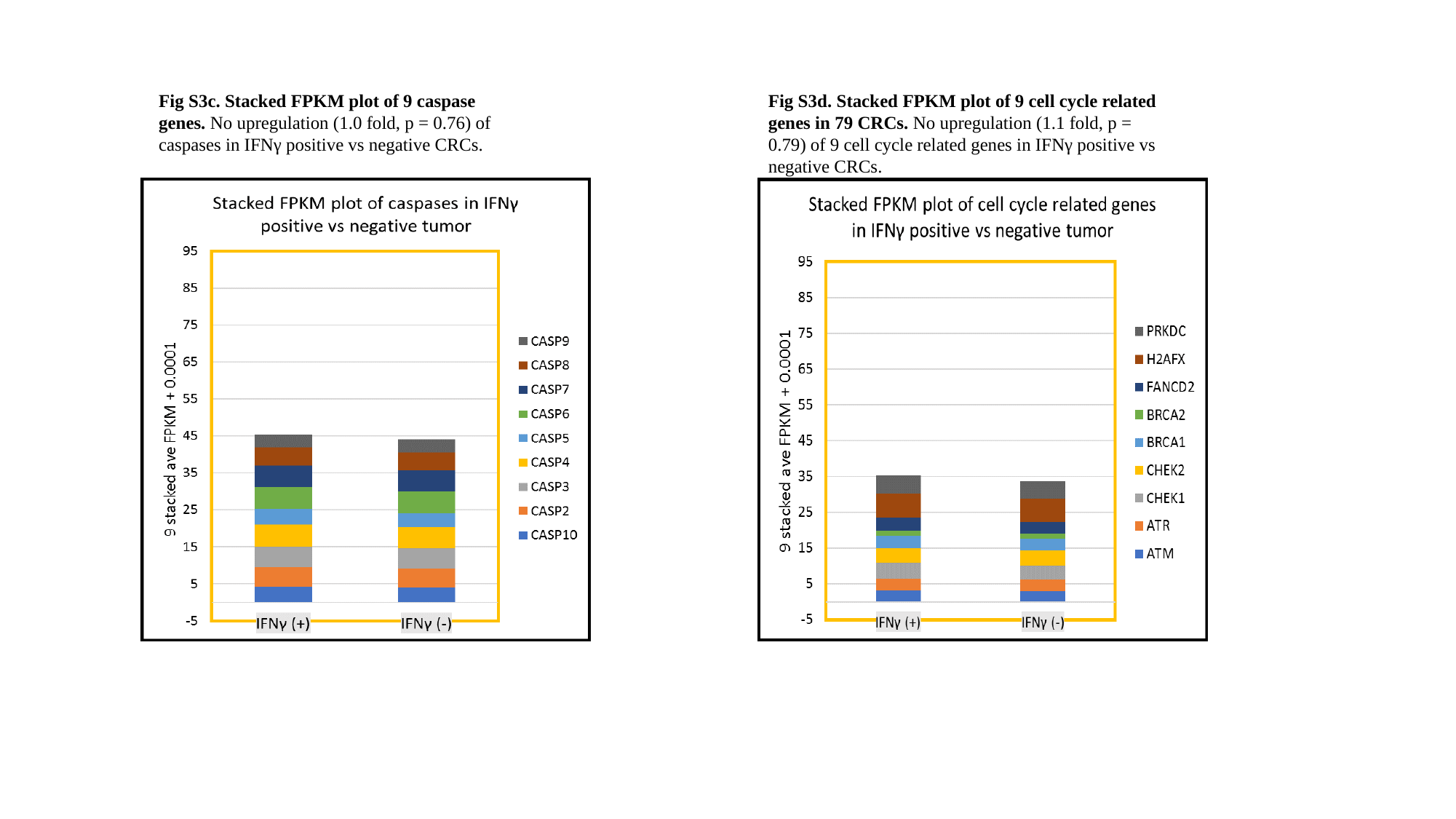

Fig S3d. Stacked FPKM plot of 9 cell cycle related genes in 79 CRCs. No upregulation (1.1 fold, p = 0.79) of 9 cell cycle related genes in IFNγ positive vs negative CRCs.
Fig S3c. Stacked FPKM plot of 9 caspase genes. No upregulation (1.0 fold, p = 0.76) of caspases in IFNγ positive vs negative CRCs.

## Slide 19
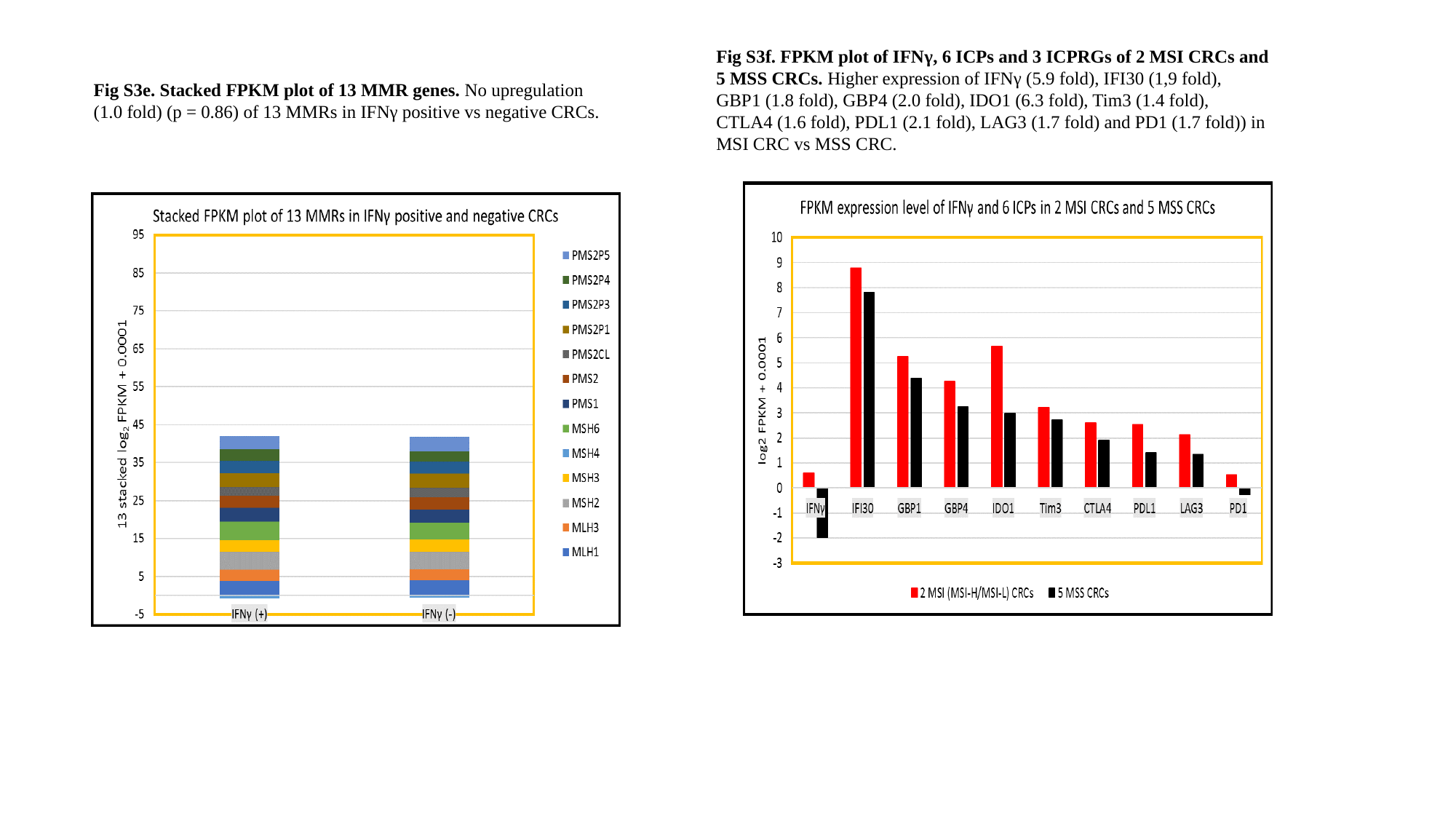

Fig S3f. FPKM plot of IFNγ, 6 ICPs and 3 ICPRGs of 2 MSI CRCs and 5 MSS CRCs. Higher expression of IFNγ (5.9 fold), IFI30 (1,9 fold), GBP1 (1.8 fold), GBP4 (2.0 fold), IDO1 (6.3 fold), Tim3 (1.4 fold), CTLA4 (1.6 fold), PDL1 (2.1 fold), LAG3 (1.7 fold) and PD1 (1.7 fold)) in MSI CRC vs MSS CRC.
Fig S3e. Stacked FPKM plot of 13 MMR genes. No upregulation (1.0 fold) (p = 0.86) of 13 MMRs in IFNγ positive vs negative CRCs.

## Slide 20
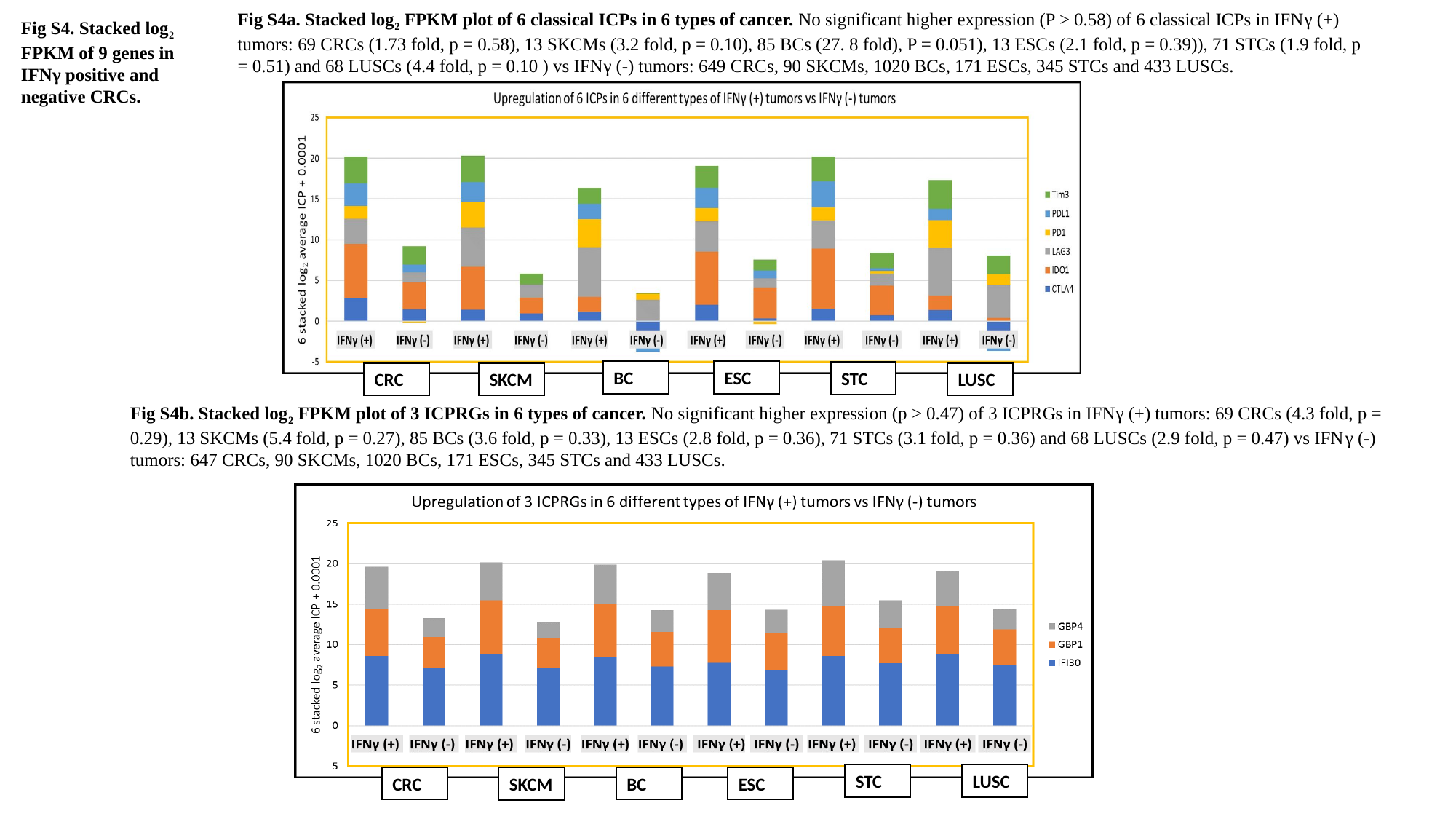

Fig S4a. Stacked log2 FPKM plot of 6 classical ICPs in 6 types of cancer. No significant higher expression (P > 0.58) of 6 classical ICPs in IFNγ (+) tumors: 69 CRCs (1.73 fold, p = 0.58), 13 SKCMs (3.2 fold, p = 0.10), 85 BCs (27. 8 fold), P = 0.051), 13 ESCs (2.1 fold, p = 0.39)), 71 STCs (1.9 fold, p = 0.51) and 68 LUSCs (4.4 fold, p = 0.10 ) vs IFNγ (-) tumors: 649 CRCs, 90 SKCMs, 1020 BCs, 171 ESCs, 345 STCs and 433 LUSCs.
Fig S4. Stacked log2 FPKM of 9 genes in IFNγ positive and negative CRCs.
BC
ESC
STC
CRC
SKCM
LUSC
Fig S4b. Stacked log2 FPKM plot of 3 ICPRGs in 6 types of cancer. No significant higher expression (p > 0.47) of 3 ICPRGs in IFNγ (+) tumors: 69 CRCs (4.3 fold, p = 0.29), 13 SKCMs (5.4 fold, p = 0.27), 85 BCs (3.6 fold, p = 0.33), 13 ESCs (2.8 fold, p = 0.36), 71 STCs (3.1 fold, p = 0.36) and 68 LUSCs (2.9 fold, p = 0.47) vs IFNγ (-) tumors: 647 CRCs, 90 SKCMs, 1020 BCs, 171 ESCs, 345 STCs and 433 LUSCs.
STC
LUSC
CRC
BC
ESC
SKCM

## Slide 21
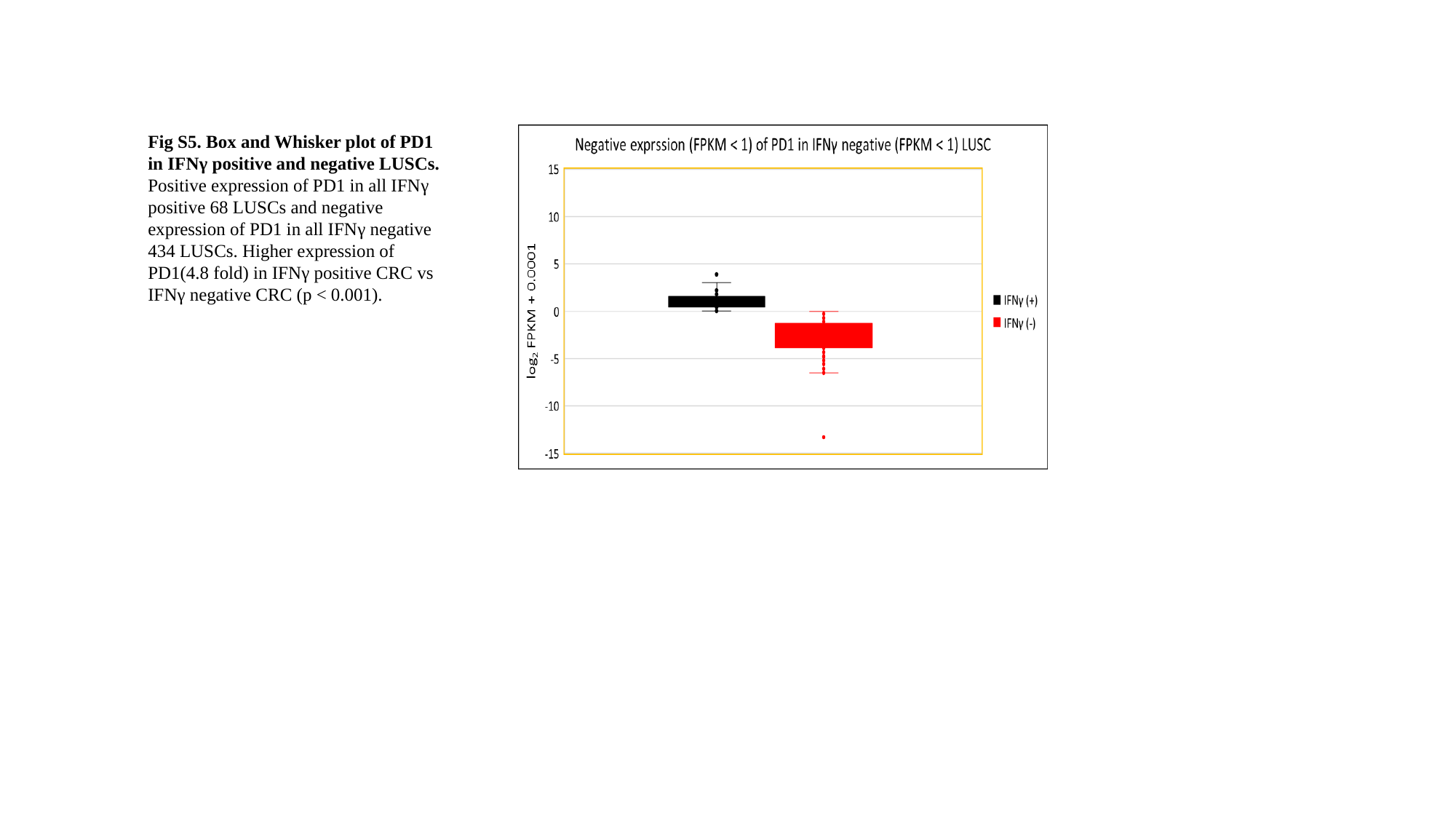

Fig S5. Box and Whisker plot of PD1 in IFNγ positive and negative LUSCs. Positive expression of PD1 in all IFNγ positive 68 LUSCs and negative expression of PD1 in all IFNγ negative 434 LUSCs. Higher expression of PD1(4.8 fold) in IFNγ positive CRC vs IFNγ negative CRC (p < 0.001).

## Slide 22
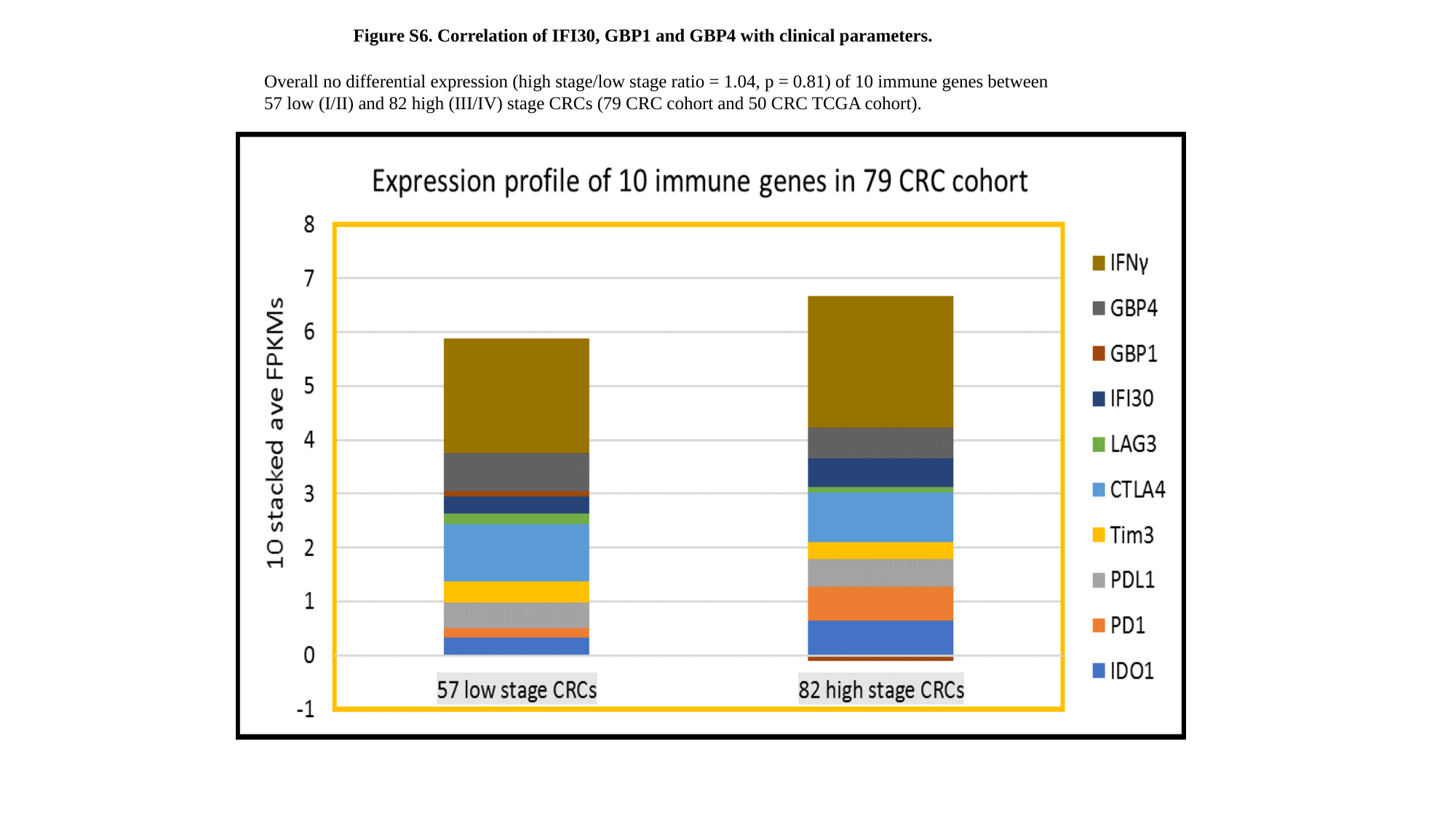

Figure S6. Correlation of IFI30, GBP1 and GBP4 with clinical parameters.
Overall no differential expression (high stage/low stage ratio = 1.04, p = 0.81) of 10 immune genes between 57 low (I/II) and 82 high (III/IV) stage CRCs (79 CRC cohort and 50 CRC TCGA cohort).

## Slide 23
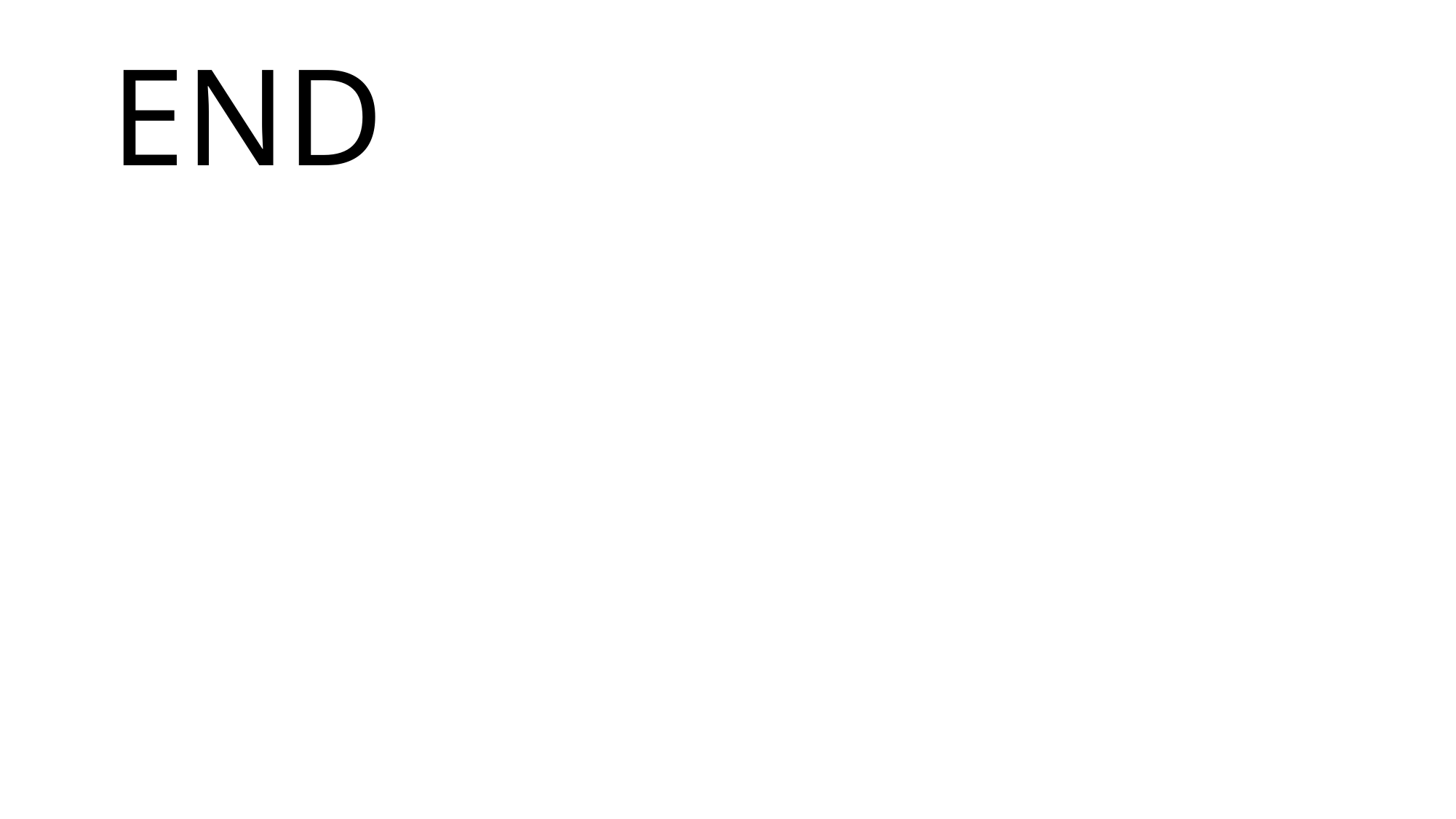

# END
